# A Phenomenological Model for Predicting Melting Temperatures of DNA Sequences

**DOI:** 10.1371/journal.pone.0012433

**Published:** 2010-08-26

**Authors:** Garima Khandelwal, Jayaram Bhyravabhotla

**Affiliations:** 1 Department of Chemistry, Indian Institute of Technology Delhi, New Delhi, India; 2 Supercomputing Facility for Bioinformatics and Computational Biology, Indian Institute of Technology Delhi, New Delhi, India; 3 School of Biological Sciences, Indian Institute of Technology Delhi, New Delhi, India; Midwestern University, United States of America

## Abstract

We report here a novel method for predicting melting temperatures of DNA sequences based on a molecular-level hypothesis on the phenomena underlying the thermal denaturation of DNA. The model presented here attempts to quantify the energetic components stabilizing the structure of DNA such as base pairing, stacking, and ionic environment which are partially disrupted during the process of thermal denaturation. The model gives a Pearson product-moment correlation coefficient (r) of ∼0.98 between experimental and predicted melting temperatures for over 300 sequences of varying lengths ranging from 15-mers to genomic level and at different salt concentrations. The approach is implemented as a web tool (www.scfbio-iitd.res.in/chemgenome/Tm_predictor.jsp) for the prediction of melting temperatures of DNA sequences.

## Introduction

Several physico-chemical factors such as base stacking, hydrogen bonding, hydrophobic, electrostatic and van der Waals interactions etc. stabilize the DNA molecule [1]. Base stacking and hydrogen bonding are considered to be the dominant of all these forces [2]–[4]. These diverse forces stabilizing DNA act in concert to protect the genetic code against external perturbations. But if these forces render the DNA to be static, the coding bases will not be directly accessible to the expression of genetic code. DNA, however, is a dynamic entity and the forces do get disrupted and the coding bases exposed to enzymes [5] as in replication of DNA, transcription into m-RNA etc.. How DNA opens up in response to intrinsic sequence effects and extrinsic local environment is thus a matter of considerable interest in deciphering molecular details of gene expression in particular and genome organization in general. We have been interested in understanding the sequence effects on the structure and energetics of DNA [6]–[8]. Here we focus on the stability of DNA of varying lengths and base composition and constitution from a melting perspective.

DNA denaturation (melting) is the process of separation of ds-DNA into two single strands. This cooperative unwinding is also known as helix-coil or melting transition [9]. DNA melting occurs over a small range of temperature and results in changes in its physical properties [10]. It has been known since the 1950s, that heating a DNA solution above room temperature results in the separation of strands. The temperature at which half of the DNA molecule is denatured, i.e. one half is in double helical form and the other half in a random coil state, is termed as the melting temperature of the DNA, Tm [9]. The melting temperature depends on a variety of factors, such as the length of DNA [11], [12] (shorter pieces tend to melt more easily, [13]), the nucleotide sequence composition [14]–[16], salt concentration (ionic strength of the added salt) [14]–[15], [17] and generally lies between 50°C and 100°C. DNA can be denatured not only by heating, but by other methods as well, eg. use of organic solvents such as formamide [18] and dimethyl sulfoxide, ligands [19], increasing the pH of the solution, lowering the salt concentration [20] etc.

DNA ‘breathes’ even at normal cell temperatures [21], [22] and local regions of a few tens of base pairs become temporarily unwound and form a bubble, in which stacking and hydrogen bonding are partially disrupted [23]–[25]. It is easier for the proteins (RNA polymerase, and origin binding proteins) to create locally unwound regions on DNA in A/T rich regions, which could be one of the reasons for DNA replication origins and transcription initiation bubbles to have such regions [26]. In G/C rich regions, the strands do not unwind until higher temperatures are reached. When all of the base interactions are broken, the two strands separate. This is called denaturation. Local unwinding however, is not denaturation but an essential prerequisite.

DNA melting is measured by the absorbance of UV light (260 nm) by the DNA solution, where the amount of UV light absorbed is proportional to the fraction of non-bonded base pairs. This UV absorbance is due to the π-π* electronic transition in both purine and pyrimidine bases, which reflects a change in the electronic configuration of the bases due to the decrease in double helical stacking and base paring upon melting. As the temperature increases, melting of the double-stranded DNA is initiated and the absorbance of UV-light increases through a series of sharp jumps. The absorbance increases by 30–40% depending on the DNA sample. [9]. The middle-point of the temperature range over which the strands of DNA separate gives the melting temperature [10].

Earlier theories on DNA melting have incorporated stacking and hydrogen bonding within the framework of models for transitions in polypeptides: (i) Zimm-Bragg theory; where stacking is modeled as a nearest-neighbor interaction; [27] (ii) Lifson-Roig theory; where conformational restriction due to hydrogen bonding is taken into account [28]. The role of stacking against the background of hydrogen bonding has been investigated within the context of Generalized Model of Polypeptide Chain (GMPC) [29]. Other descriptions of melting have also been advanced [30]–[32]. Theories addressing the helix-coil transitions are not widely used for the prediction of melting temperatures [32]. One of the reasons for this could be the difficulty in calculations, which are computation-intensive and require adjustment of many parameters [33].

Many attempts have been made to predict the melting temperatures of short nucleotide sequences, which is of particular interest in primer design. The earliest of these methods used a simple formula to calculate Tm based on the GC content of the sequence [17]. Subsequently, this formula was modified to include the effect of salt concentration of the solution [20]. The next set of methods utilized the nearest neighbor (NN) model to calculate Tm, which requires a set of thermodynamic parameters. Many groups have provided these parameters [14], [34], [35] and, it was noted that there was a consensus among these methods [35]. While the ranges of energy determined in different studies are similar, the values for individual NN pairs show discrepancies [36]. Also, the coefficients obtained by these methods from fitting the data are non-unique and defy simple interpretation [4]. Taking the research efforts a step further towards a reliable predictive model, we report in this work, a phenomenological model to predict the melting temperature of DNA, accounting for the physico-chemical events taking place in the melting process. In particular, the model introduced here accounts quantitatively and explicitly for disruption in stacking interactions, breakage of hydrogen bonding, salt effects and the nucleotide strand concentration in the melting of DNA.

## Materials and Methods

### Dataset

The accuracy benchmark dataset compiled by Panjkovich & Melo [37] is adopted here for the study. The dataset is made up of 348 data points comprising 108 unique oligonucleotide sequences at various salt concentrations. This dataset is divided into two parts: (i) A training set consisting of 123 oligomers for obtaining the best fit equation giving the minimum possible error and (ii) a test dataset consisting of 225 oligomers, to assess the quality of prediction on independent data. Both the datasets represents the complete data space (Figures S1, S2 and S3). We have also examined the performance of the method on an additional dataset of 100 short nucleotide sequences (15mers) [38]. Subsequently, we investigated the validity of the model on 20 genomic sequences.

### Methodology

Melting of DNA necessitates the disruption of stacking interactions between the two base pairs within each dinucleotide step. During the process, cross strand stacking interactions are completely lost while intra-strand stacking interactions are disrupted partially. The dinucleotide steps are assembled into four groups on the basis of their possible interactions as RR, RY, YR and YY, where R and Y denote a purine and a pyrimidine respectively. RY has the highest stacking as known from experiments [39] and simulations [40]. Various combinations of values were tried out to give the least possible error for the training dataset. Finally, the four dinucleotide groups (RY, RR, YY, YR) were assigned values as 5, 3, 3, 2, keeping in mind that the values should be relative to the values for H-bonding as well as to each other.

The melting of DNA also requires the breakage of Watson-Crick hydrogen bonds (H-bonds) and it is well known that GC pairs (3 H-bonds) are stronger than AT pairs (2 H-bonds). Based on this, and the knowledge of interaction energies of H-bonded pairs [7], [41], values of 4 and 1 are assigned to GC and AT base pairs respectively. On the basis of hydrogen bonding between the bases, the double helical dinucleotide steps can be divided into three groups: (a) Group with 6 H-bonds, (b) Group with 5 H-bonds and (c) Group with 4 H-bonds; the corresponding H-bond energy values being 8, 5 and 2 respectively.

The contribution of H-bond energy and stacking energy is almost equivalent in the stabilization of duplex DNA, as discerned from various studies on modified bases [42], and dangling bases [39] and is of the order of 1–2 kcal. Also, it has been observed that the rise in melting temperature due to the addition of a single H-bond is about 2–6°C [43], while it is approximately 2°C due to increase in stacking energy per added base pair [44]. The H-bonding and stacking energy values are assigned considering all these observations. The DNA strength parameter for each double helical dinucleotide step can be then developed as a sum of stacking and hydrogen bonding values proposed above. For example, in case of GC, which belongs to RY group, the value of stacking is 5 while two triple H-bonds add up to a value of 8. So, the DNA strength parameter for a GC step is given as: 5+8 = 13.

A total of 16 dinucleotide combinations are possible of which only 10 are unique when read in the 5′ → 3′ direction. These are arranged here in the decreasing order of DNA strength parameter value (Table1): (i) GC, (ii) CC = GG, (iii) CG, (iv) AC = GT, (v) TC = GA, (vi) CT = AG, (vii) TG = CA, (viii) AT, (ix) TT = AA, (x) TA. The above assignment of DNA strength parameter values is also found to be consistent with the observations on relative stabilities of dinucleotides [25], the molecular interpretation of the conjugate rule [45] and some recent molecular dynamics simulations [40]. These values are found to be in overall agreement with the calculated free energies [14], [15], [34], [46] and melting free energy parameters [36] with a few exceptions.

The value of DNA strength parameter for the whole sequence is accumulated by adding the values (Table 1) for each dinucleotide step which is referred to here as the cumulative DNA strength parameter. This would go on increasing with the length, so to delineate the effect of length, the DNA strength parameter (E) is derived on a per unit (base pair) basis as given below:
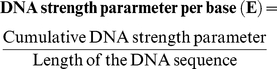



**Table 1:** Values of DNA strength parameter for each dinucleotide step.

Stack	5	3	3	2
H-bond	RY	YY	RR	YR
4+4	GC = 13	CC = 11	GG = 11	CG = 10
1+4	AC = 10	TC = 8	AG = 8	TG = 7
4+1	GT = 10	CT = 8	GA = 8	CA = 7
1+1	AT = 7	TT = 5	AA = 5	TA = 4

The salt effects are taken into account on the basis of [Na^+^] concentration in the solution, implemented as a natural logarithmic variable, which is in accordance with previous work [38], [47]. Similarly borrowing from the electrostatic behavior of DNA from the literature [6], the length of the sequence is also accounted for via a natural logarithmic function. Length considerations via a variable such as (n−1)/n, (n  =  length of oligonucleotide sequence) were reported earlier to account for the decrease in Tm with decreasing length of the oligomer [47]. The concentration units for oligonucleotides and genomic sequences are typically reported as molar and µg/ml respectively in experimental studies. The nucleotide strand concentration parameter is implemented using a natural logarithmic function.

All the above contributors are pooled into a simple equation and processed through the multiple regression analysis method of Analyse-It software package [48], to derive the best fitting equation predicting the Tm values for the training dataset. Residual values and the standard error of estimate were also calculated. The good-ness of fit is critically evaluated by various statistical techniques such as the normal probability plots of residual, residual distribution plots (Figures S4 and S5 respectively). The final equation derived after the multiple regression is:
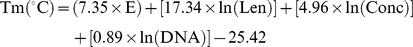
(1)


Tm  =  Predicted melting temperature

E  =  DNA strength parameter per base

Len  =  Length of nucleotide sequence (number of base pairs)

Conc  =  [Na^+^] concentration of the solution (Molar)

DNA  =  Total nucleotide strand concentration.

The r^2^ obtained from this equation on the training dataset is 0.96. The equation to predict the melting temperature, without the use of nucleotide strand concentration (DNA) as one of the parameters is provided in the supporting information (Supporting Text S1).

The use of eq. (1) is illustrated below. Consider for example a 15 bp long sequence GACGACAAGACCGCG, taken at 0.22 M salt concentration and 0.000002 nucleotide strand [38]. The melting temperature for this sequence is calculated as follows.

**Step 1:** Read the sequence from 5′ end to 3′ end and add up the DNA strength parameter given in Table 1 for each dinucleotide step, moving one base at a time as: GA = 8, AC = 10, CG = 10, GA = 8, AC = 10, CA = 7 and so on. (For the given sequence of 15 base pairs, 14 dinucleotide steps are obtained). So, The DNA strength parameter for the given sequence is: 8+10+10+8+10+7+5+8+8+10+11+10+13+10 = 128. The DNA strength parameter per base (E) is then calculated as: 128/15 = 8.53

**Step 2:** Substituting all the values in eq. (1),



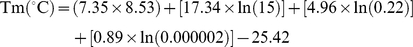

**Predicted Tm  = 65.04°C**

**Reported Experimental Tm  = 64.4°C [38]**

For genomic sequences, the Tm is first calculated by computing the cumulative strength parameter of a melting unit of 70 bp from the start which is then derived per base and employed in eq. (1). This window is translated by one base pair and a new Tm is calculated and the procedure is repeated till the end of the sequence. The Tm for the whole genomic sequence is then developed as the average of overlapping melting units of length 70 bp, a number arrived at empirically which appears to have biological significance as discussed below.

## Results and Discussion

**Figure 1:**
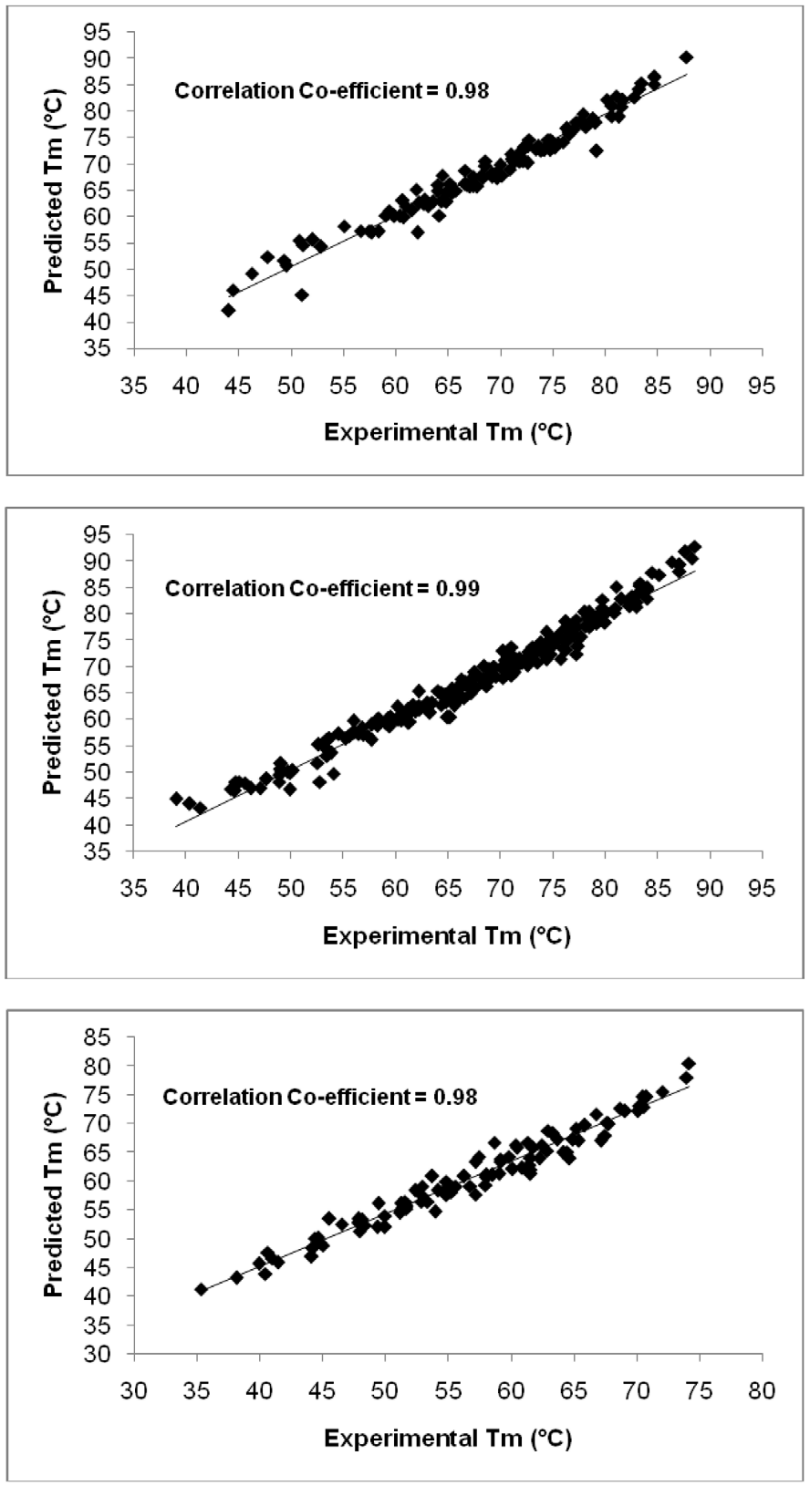
Correlation plots between the experimental and predicted melting temperatures. **Figure 1**(**a**). Correlation between predicted and experimental melting temperatures for the training dataset of 123 oligomers **Figure 1**(**b**) **.** Correlation between predicted and experimental melting temperatures for the test dataset of 225 oligomers **Figure 1**(**c**) **.** Correlation between predicted and experimental melting temperatures for an additional dataset of 100 oligomers (15-mers) adapted from Ref. 38.

In this study, a phenomenological model is developed on the basis of a theoretical appraisal of the events occurring during the process of DNA thermal denaturation. The model was trained on a dataset of 123 oligomers to achieve a best fit equation (1); (Figure 1), which gave a correlation coefficient (r) of 0.98 and an average error of 1.36°C (data provided in Table S1). This equation (1) was used to predict the melting temperatures for a test dataset of 225 oligonucleotide sequences whose experimental melting temperatures were known; (Figure 1), where a correlation coefficient (r) of 0.99 and an average error of 1.31°C was obtained (data provided in Table S2). Subsequently the model was validated on 100 15-mers compiled by Owczarzy [38]. The results are depicted in Fig. 1(c), which indicate that even for shorter sequences not occurring in the training set, the correlation between the predicted and the experimental Tm on a large dataset of 100 sequences is quite high (correlation coefficient, r = 0.98, data provided in Table S3). A further verification of the viability of the current method was undertaken by considering three oligonucleotide sequences of 40 base pair length, taken at two different salt concentrations [38]. The average error of prediction for these sequences is 1.48°C (data provided in Table S4). The significance of the model was checked by means of Anova (Table S5).

The correlation coefficients with experimental melting temperatures for the four parameters used in the model, as a single entity and in all possible combinations are shown in Table 2. As clear from Table 2, the strength parameter appears to be the main driving force in the melting of DNA. The length of the nucleotide sequence as well as the concentration of the solution also play a substantial role in the melting of DNA, where the effect of concentration is more pronounced than that of length when combined with the strength parameter, but even both of them together do not reach up to the mark of strength parameter taken alone. Although the correlation achieved after adding the strand concentration (DNA) does not improve much, the average error between the experimental and predicted Tm comes down marginally; hence it is retained in the model.

**Table 2:** Correlation coefficients for all possible combinations of the four parameters used in eq. (1).

Parameter	Correlation Coefficient (r)
E	0.77
Len	0.49
Conc	0.44
DNA	−0.21
E + Len	0.83
E + Conc	0.93
E + DNA	0.71
Len + Conc	0.65
Len + DNA	0.49
Conc + DNA	0.50
E + Len + Conc	0.98
E + Len + DNA	0.84
E + Conc + DNA	0.93
Len + Conc + DNA	0.66
E + Len + Conc + DNA	0.98

E  =  DNA strength parameter per base; Len  =  Length of nucleotide sequence (number of base pairs); Conc  =  [Na+] concentration of the solution (Molar); DNA  =  Total nucleotide strand concentration (Molar).

The following methods were reported earlier in the literature for melting temperature predictions: (i) Basic method [17]; (ii) Salt corrected method [20]; (iii) NN method using Breslauer's parameters [14]; (iv) NN method using Santa Lucia's parameters [35]; (v) NN method using Sugimoto's parameters [34] and (vi) Consensus method [37]. On the basis of a previous comparison of various Tm prediction methods, it was observed that the best methods were the Nearest Neighbor methods based on thermodynamic properties, but the major drawback with these methods was that they applied well primarily to oligomers ranging from 4 to 20 bp [37]. Panjkovich and Melo [37] after an extensive study, observed that under certain experimental conditions of salt and oligonucleotide concentration, even a very simple method that did not take into account these parameters could give results similar to the more complex methods, but under variable salt and oligonucleotide concentrations, the thermodynamic methods outperformed the simpler ones. We infer from the results presented here that a simple model [eq. (1)] developed on the basis of a quantification of forces destabilized during melting shows satisfactory performance for any length of the oligonucleotide sequence, salt concentration and base composition.

### Extension of the methodology to genomes

The melting temperatures of 20 genomes were also calculated using eq. (1) as described in the methods section. The results are compared with the experimental data [12], [13], [49], [50] and presented in Table 3.

**Table 3:** Experimental and predicted melting temperatures of a few genomic DNA sequences.

S. No.	Genome	NCBI ID	Length (bp)	Na ^+^ Conc. (M)	DNA Conc. (g/ml)	Exp. Tm (°C)	Pred. Tm (°C)	Exp. – Pred. Tm(°C)
1.	*Cytophaga hutchinsonii*	NC_008255	4433218	0.016	0.00002	70.2[^49^]	73	−2.8
2.	*Lactobacillus acidophilus*	NC_006814	1993560	0.016	0.00002	67.9[^49^]	71.1	−3.2
3.	*Lactobacillus bulgaricus*	NC_008054	1864998	0.016	0.00002	74.9[^49^]	77.6	−2.7
4.	*Lactobacillus fermenti*	NC_010610	2098685	0.016	0.00002	75.6[^49^]	78.4	−2.8
5.	*Leptospira interrogans*	NC_004343	358943	0.016	0.00002	68.4[^49^]	71.1	−2.7
6.	*Leptospira borgpetersenii*	NC_008508	3614446	0.016	0.00002	72.4[^49^]	73.3	−0.9
7.	*Mycoplasma arthritidis*	NC_011025	820453	0.016	0.00002	65.9[^49^]	69.3	−3.4
8.	*Micrococcus luteus*	NC_012803	2501097	0.016	0.00002	84.9[^49^]	87.9	−3
9.	*Nitrobacter winogradskyi*	NC_007406	3402093	0.016	0.00002	81.0[^49^]	83.2	−2.2
10.	*Pseudoalteromonas atlantica*	NC_008228	5187005	0.016	0.00002	71.2[^49^]	75.6	−4.4
11.	*Pseudomonas pseudomallei*	NC_006350	4074542	0.016	0.00002	84.3[^49^]	85.8	−1.5
12.	*Stenotrophomonas maltophilia*	NC_010943	4851126	0.016	0.00002	83.1[^49^]	85.2	−2.1
13.	*Pseudomonas fluorescens*	NC_004129	7074893	0.016	0.00002	80.1[^49^]	83.7	−3.6
14.	*Shewanella putrefaciens*	NC_009438	4659220	0.016	0.00002	73.2[^50^]	75.5	−2.3
15.	*Bacillus subtilis*	NC_000964	4214630	0.0732	0.00005	82.1 [^12^]	83.3	−1.2
16.	*Clostridium perfringens*	NC_003366	3031430	0.0732	0.00005	75.1[^12^]	76.7	−1.6
17.	*Micrococcus luteus*	NC_012803	2501097	0.0732	0.00005	94.5[^12^]	96.3	−1.8
18.	*Pseudomonas fluorescens*	NC_004129	7074893	0.0732	0.00005	89.8[^12^]	92.1	−2.3
19.	*Bacillus subtilis*	NC_000964	4214630	0.15	0.00002	87[^13^]	86	1
20.	*Deinococcus radiodurans*	NC_001263	2648638	0.15	0.00002	97[^13^]	96.4	0.6
21.	*Mycobacterium leprae*	NC_002677	3268203	0.15	0.00002	93[^13^]	92.5	0.5
22.	*Saccharomyces cerevisiae*	NC_001133 to NC_001148	12057500	0.15	0.00002	82.5[^13^]	83.8 ^Ω^	−1.3
23.	*Ureaplasma urealyticum*	NC_011374	874478	0.15	0.00002	78[^13^]	78.4	−0.4

Ω Average melting temperature for the 16 chromosomes.

Exp. Tm  =  Experimental melting temperature.

Pred. Tm  =  Predicted melting temperature.

The melting of large and genomic level sequences can be modeled as a cooperative phenomenon, occurring simultaneously at various places along the DNA sequence, where each melting region can be described as a “melting unit” [51]. The size of the melting unit has been a centre of attention for many years. Many estimates have been provided in the literature on the size of the unit specific to a given sequence [52]–[53], but there has been no molecular level explanation towards the number of base pairs present in a melting unit. Moreover, the size of the melting unit estimated is highly variable. We have investigated the melting temperature for large DNA sequences in terms of melting units of various sizes ranging from 40 bp all the way upto 100 bp and found the predictions to converge well for units of size 60–70 base pairs. Thus a choice of 70 base pairs as a melting unit is made in this study. This is also found to be in accord with the literature regarding packaging of DNA in a compact form with the help of bacterial HU proteins (58 bp [54]), archaeal histones (60 bp [55]; 80 bp [56]) and eukaryal histones (70 bp [54]; 70 bp [57]). These proteins adapt themselves to open the double stranded DNA into single stranded DNA, forming a bubble of approximately the same length as the melting unit, to perform the necessary molecular tasks such as transcription [54]–[56] and replication of DNA. Our choice (hypothesis) of 70 base pairs seems to be validated by the results presented in Table 3 where the correlation between experimental and predicted values is excellent (correlation coefficient, r = 0.98; average error of prediction  = 2.0°C). The last column of Table 3 depicting the difference between experimental and predicted melting temperatures does not show any obvious pattern.

The melting temperatures of *Escherichia coli* at various salt concentrations are calculated and reported in Table 4. It may be seen from the 1^st^ entry (Experimental Tm  = 70.7°C) and the 2^nd^ entry (Experimental Tm  = 75.7°C) of the table that there are discrepancies in the experimental melting temperature values derived by various methods at nearly the same salt and nucleotide concentrations. Allowing for this difference, it may be noted that the calculations are in general accord with experiment.

**Table 4:** Experimental and predicted melting temperatures of *Escherichia coli* DNA at various salt concentrations.

S. No.	Genome	Na^+^ Conc. (M)	DNA Conc. (g/ml)	Experimental Tm (°C)	Predicted Tm (°C) #	Experimental – Predicted Tm (°C)
1.	*Escherichia coli*	0.015	0.000018	70.7[^20^]	77.9	−7.2
2.	*Escherichia coli*	0.016	0.00002	75.7[^49^]	78.3	−2.6
3.	*Escherichia coli*	0.0732	0.00005	85.7[^12^]	86.6	−0.9
4.	*Escherichia coli*	0.075	0.000018	83.3[^20^]	85.9	−2.6
5.	*Escherichia coli*	0.01	0.000018	68.7[^20^]	75.8	−7.1
6.	*Escherichia coli*	0.02	0.000018	73.4[^20^]	79.3	−5.9
7.	*Escherichia coli*	0.035	0.000018	77.1[^20^]	82.1	−5
8.	*Escherichia coli*	0.05	0.000018	80.0[^20^]	83.8	−3.8
9.	*Escherichia coli*	0.1	0.000018	86.5[^20^]	87.3	−0.8
10.	*Escherichia coli*	0.12	0.000018	86.0[^20^]	88.2	−2.2
11.	*Escherichia coli*	0.195	0.000018	88.7[^20^]	90.6	−1.9
12.	*Escherichia coli*	0.6	0.000018	93.9[^20^]	96.2	−2.3

# *Escherichia coli* K-12 genome sequence (4639675 base pairs) obtained from NCBI (NC_000913) is used for these calculations.

In a nutshell, the phenomenological model presented here for melting temperature prediction covers a large range of salt concentration, GC content and length of DNA sequence and could pave the way for a deeper molecular-level understanding of DNA melting.

### Potential application of the methodology to genome annotation

**Figure 2:**
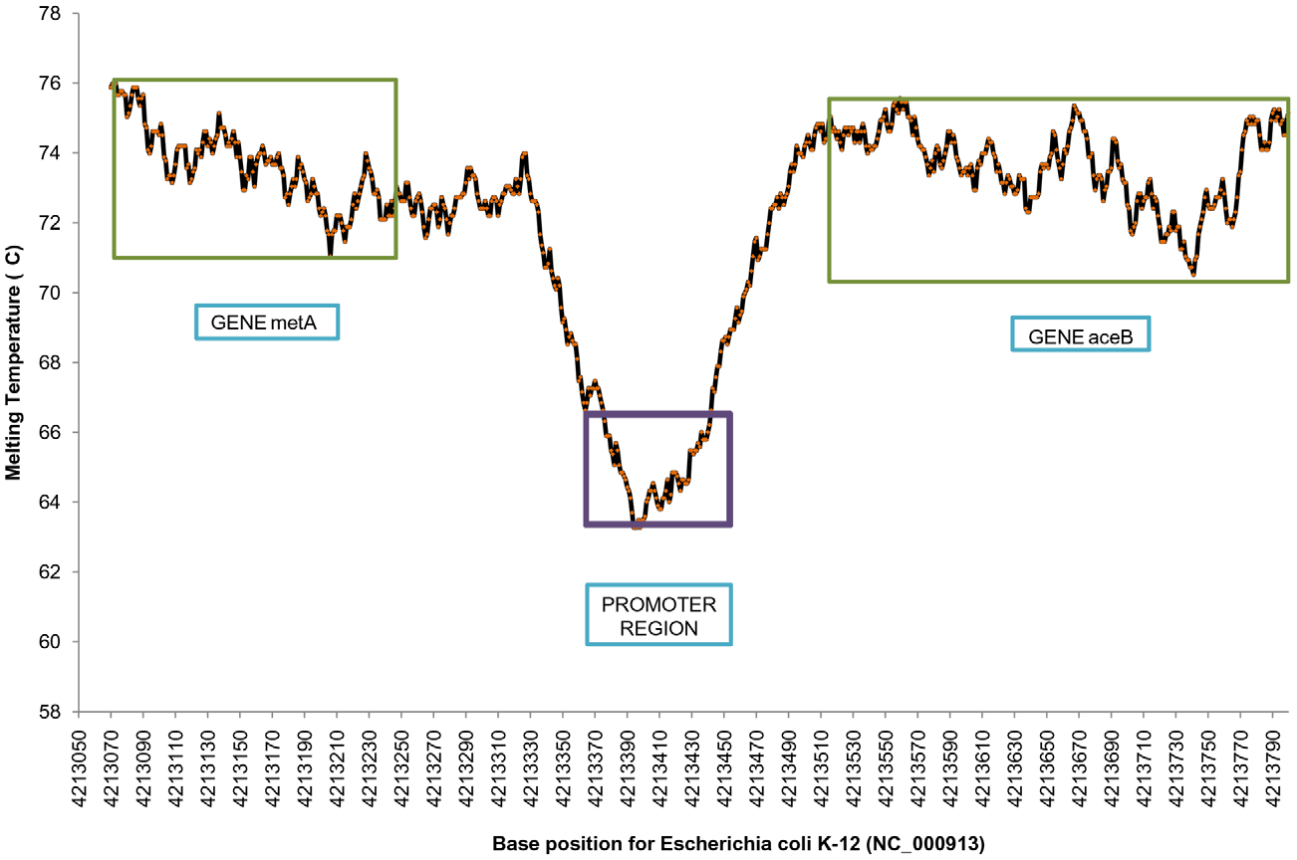
Melting profile of a promoter and its flanking genes. Melting profile for a stretch of 731 base pairs containing a promoter sequence from Ref. 59 and its corresponding experimentally verified gene sequence for *Escherichia coli* K-12 genome (NC_000913).

**Figure 3:**
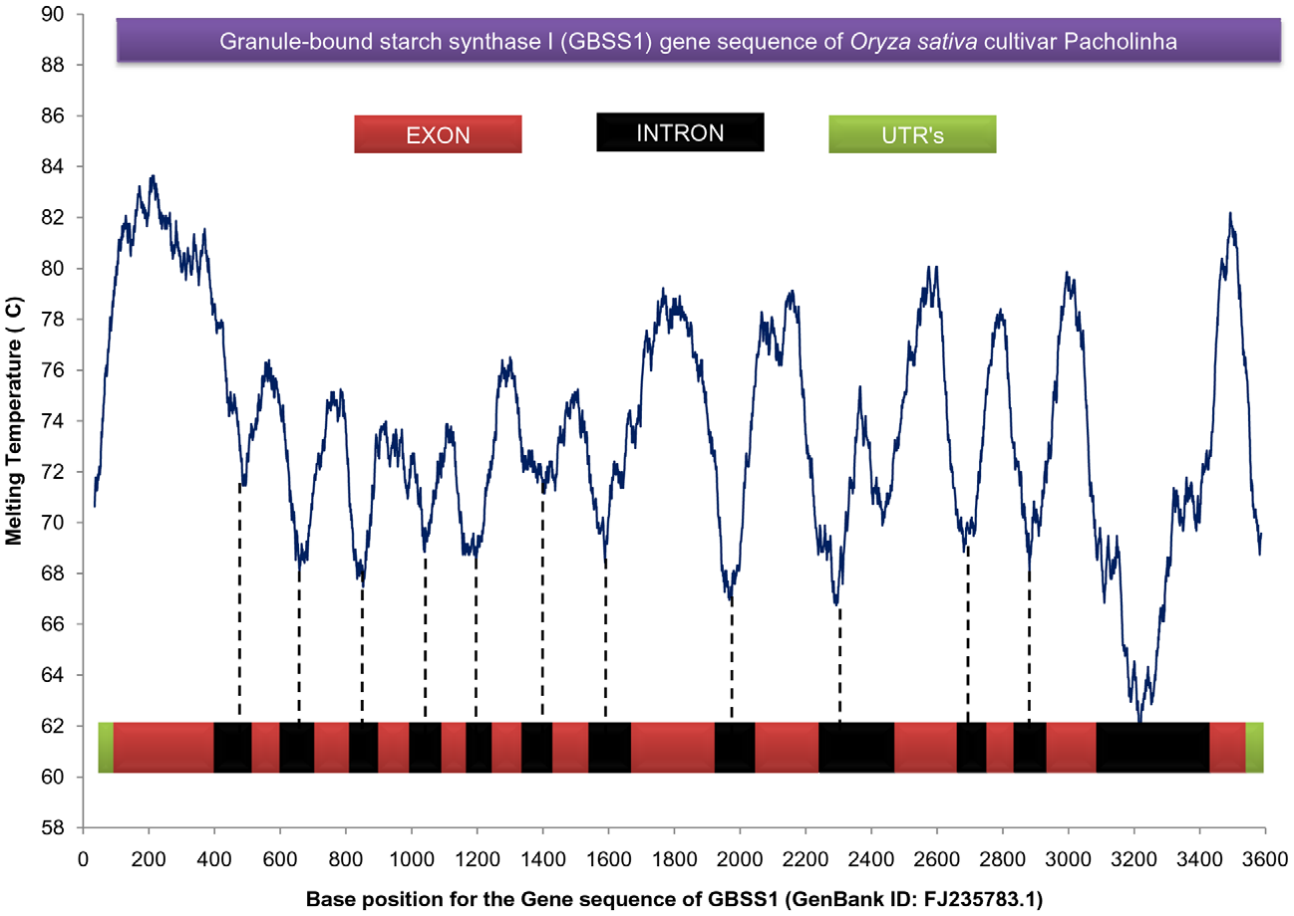
Melting profile of an *Oryza sativa* gene. Melting profile of Granule bound starch synthase I (GBSS1) gene (Length  = 3621 base pairs) *of Oryza sativa* cultivar Pacholinha (GenBank ID: FJ235783.1), showing a clear discrimination of exons from introns and Un-translated regions (UTR's).

Previous work has shown that there appears to be an underlying energy basis for the discrimination of genic and non-genic regions in prokaryotic genomes [57], [58]. As the proposed model of Tm prediction is based on the energetics of DNA, it is tempting to examine the melting temperature variations (Tm profiles) along genomic sequences. An illustrative genome profile of a part (4213070–4213801 bp) of *Escherichia coli* genome (NC_000913) is depicted in Figure 2, where a promoter region [59] is clearly differentiated from the gene region. The Tm profile of a gene (GBSS1, Gene Id: FJ235783.1) of *Oryza sativa* is shown in Figure 3, which shows discrimination of the exonic and intronic regions. Thus the methodology shows the ability to discriminate various functional units present on a genome sequence. The lower melting temperature of promoter regions could be due to the requirement of structural adaptation by DNA to facilitate specific binding of regulatory proteins, while the lower melting temperatures of introns relative to corresponding exons might be due to their low thermodynamic stability, as also observed independently by Wada and Suyama two and half decades ago [60]. Clearly, further investigations are required to utilize the strength of the methodology for genome annotation.

### Description of the web utility

The melting temperature prediction method presented here is also presented by means of a web utility: www.scfbio-iitd.res.in/chemgenome/Tm_predictor.jsp. The utility has an input box wherein the user can paste the sequence. Alternatively, the user can input the sequence with the help of buttons provided in the utility. In case of large DNA sequences, the user can also upload the sequence file through the browse option provided. The calculated Tm is reported either on the web page (for smaller sequences) or on the email-id provided by the user (for large sequences). The utility also provides the option of calculating melting temperatures at various salt and DNA concentrations. The training and test datasets and a tutorial to calculate Tm for a small sequence manually are also provided.

### Conclusion

A simple phenomenological model is developed for predicting the melting temperatures of DNA sequences based on stacking and hydrogen bonding interactions, length of the sequence, salt and nucleotide strand concentration. The model is applicable to a wide range of sequence lengths including genomic sequences, base composition and salt concentrations. This method thus overcomes the limitations noted earlier of predictive models giving good results in a limited sequence and length data space and smaller range of salt concentration. Work is in progress to develop melting profiles of complete genomes in pursuit of genome annotation to eventually facilitate a molecular level understanding of genome organization.

## Supporting Information

Figure S1 Data space representation for the length parameter.
(0.25 MB TIF)

Figure S2 Data space representation for the salt concentration parameter.
(0.23 MB TIF)

Figure S3 Data space representation for the %GC content of the sequence.
(0.48 MB TIF)

Figure S4 Normal probability plot of residuals for the training dataset.
(0.22 MB TIF)

Figure S5 Distribution of residuals with the predicted melting temperatures.
(0.37 MB TIF)

Text S1 The equation to predict the melting temperature of DNA without the use of the nucleotide strand concentration.
(0.02 MB DOC)

Table S1 Experimental and predicted melting temperatures for the training dataset of 123 oligomers.
(0.22 MB DOC)

Table S2 Experimental and predicted melting temperatures for the test dataset of 225 oligomers.
(0.38 MB DOC)

Table S3 Experimental and predicted melting temperatures for a dataset of 15-mers.
(0.16 MB DOC)

Table S4 Experimental and predicted melting temperatures for 40 base pair long oligonucleotide sequences.
(0.03 MB DOC)

Table S5 Analysis of variance for the regression equation (1) derived from the training dataset.
(0.03 MB DOC)
