# Supplementary figures and images for: A Phenomenological Model for Predicting Melting Temperatures of DNA Sequences
Source: PLoS One. 2010 Aug 26;5(8):e12433. doi: 10.1371/journal.pone.0012433 (PMC2928768; doi:10.1371/journal.pone.0012433)

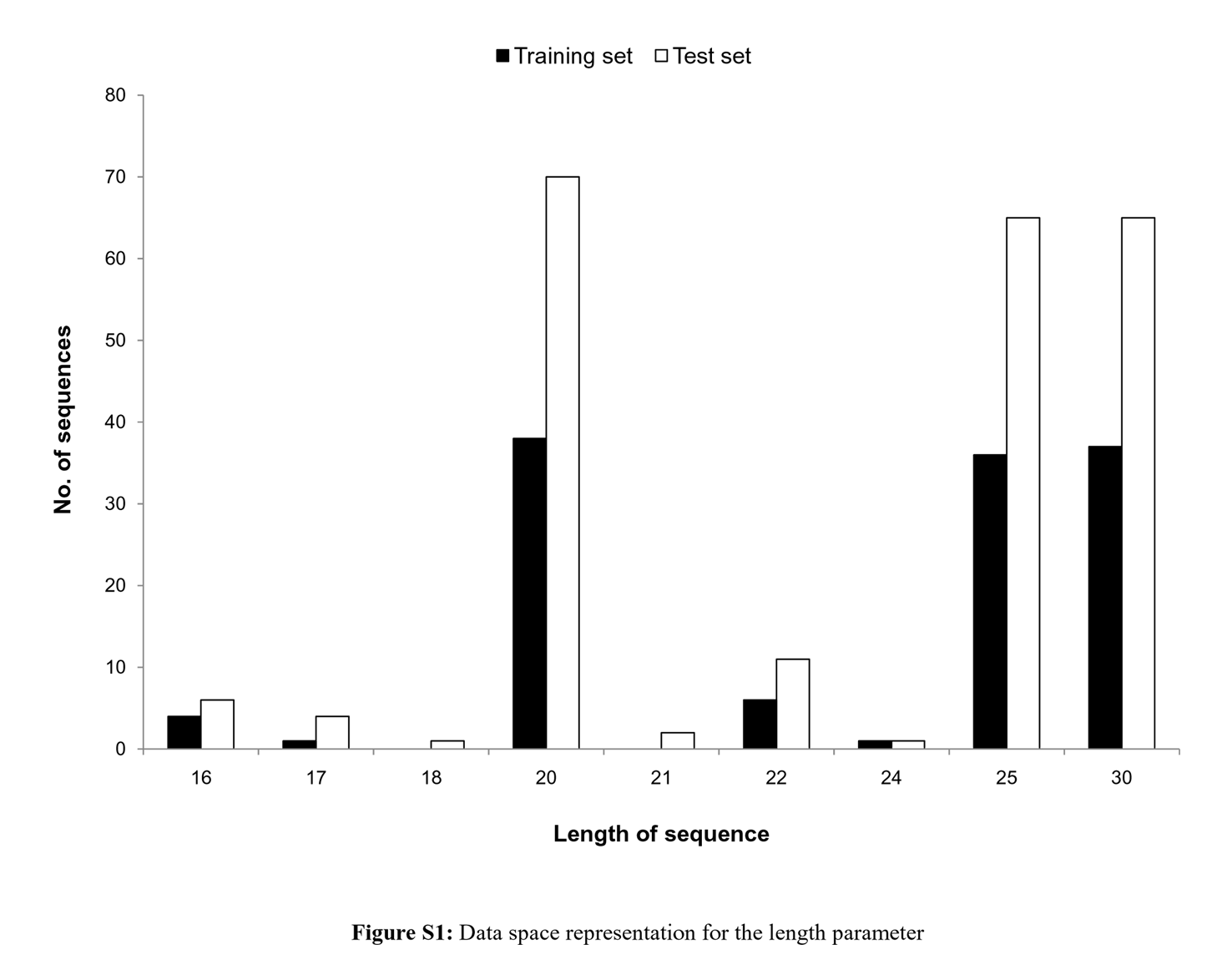

Supplement: Figure S1 — Data space representation for the length parameter. (0.25 MB TIF) [file pone.0012433.s001.tif]

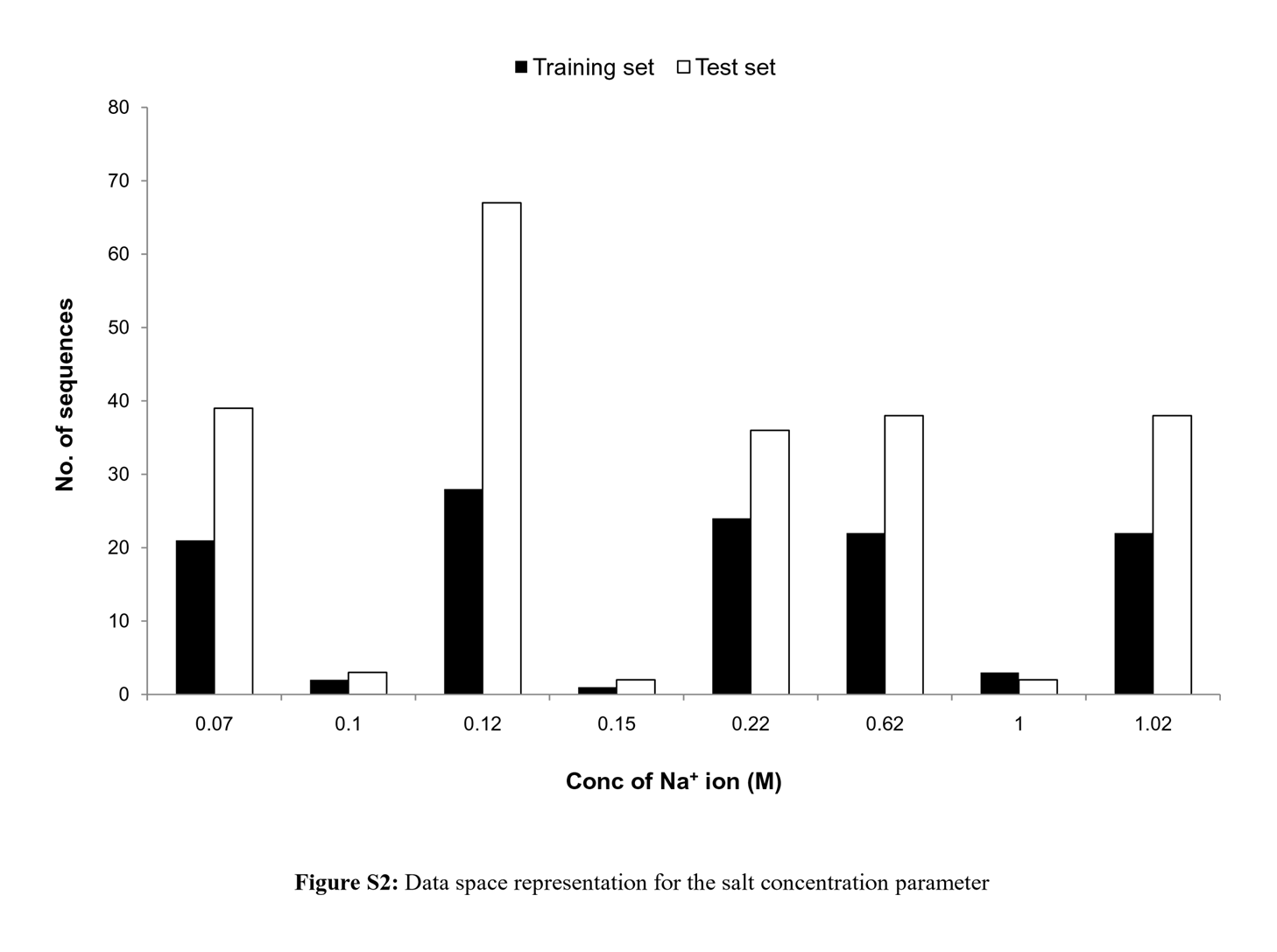

Supplement: Figure S2 — Data space representation for the salt concentration parameter. (0.23 MB TIF) [file pone.0012433.s002.tif]

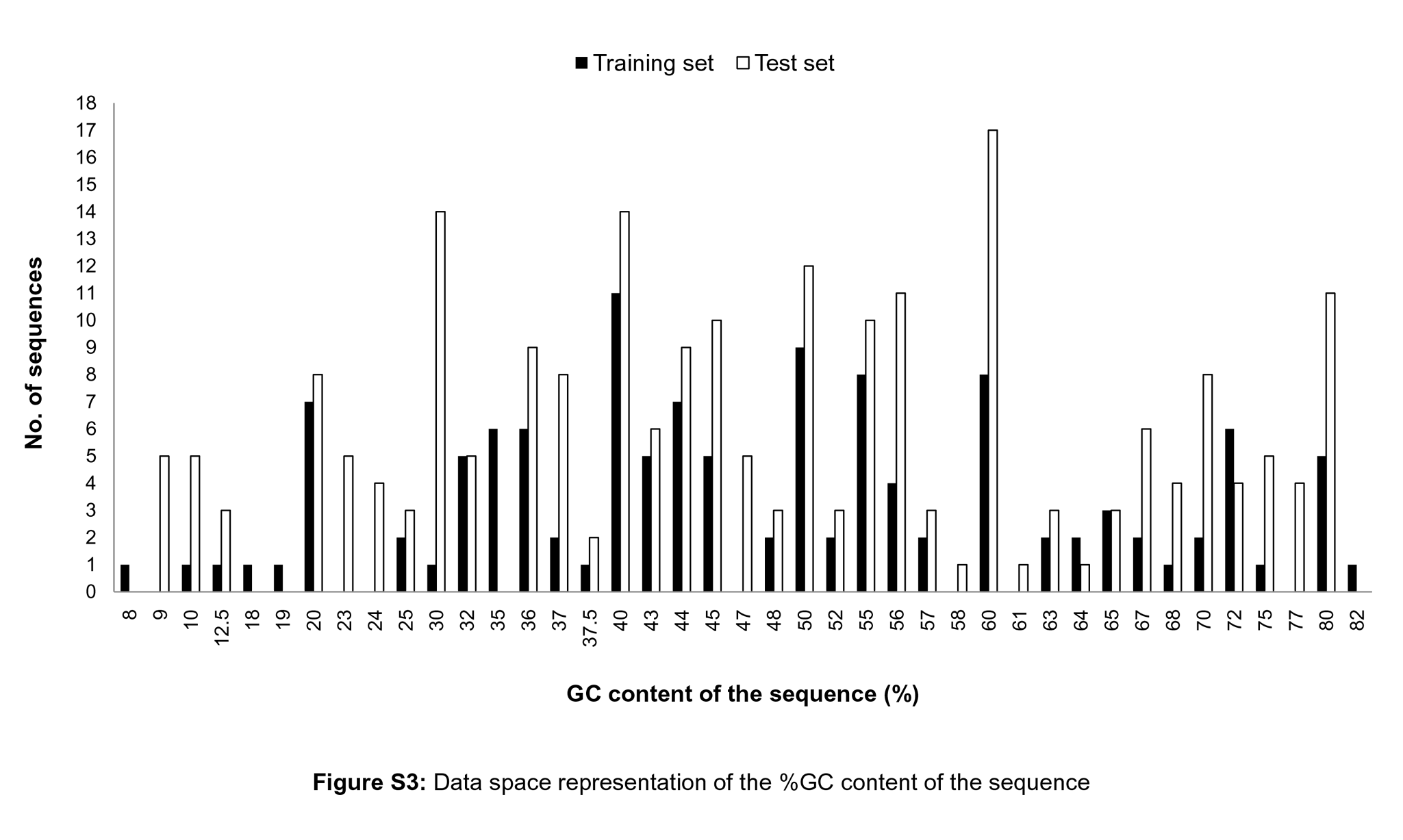

Supplement: Figure S3 — Data space representation for the %GC content of the sequence. (0.48 MB TIF) [file pone.0012433.s003.tif]

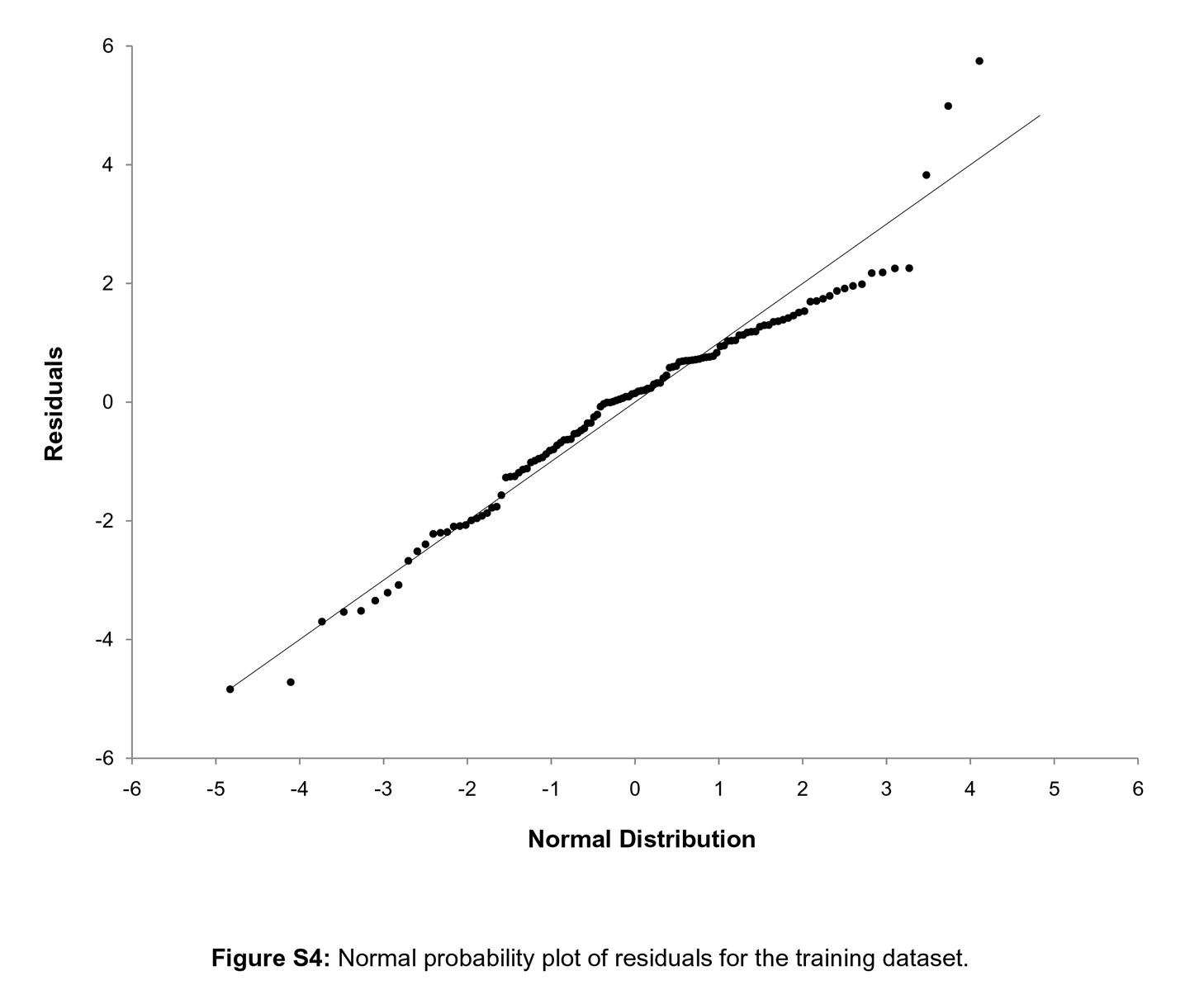

Supplement: Figure S4 — Normal probability plot of residuals for the training dataset. (0.22 MB TIF) [file pone.0012433.s004.tif]

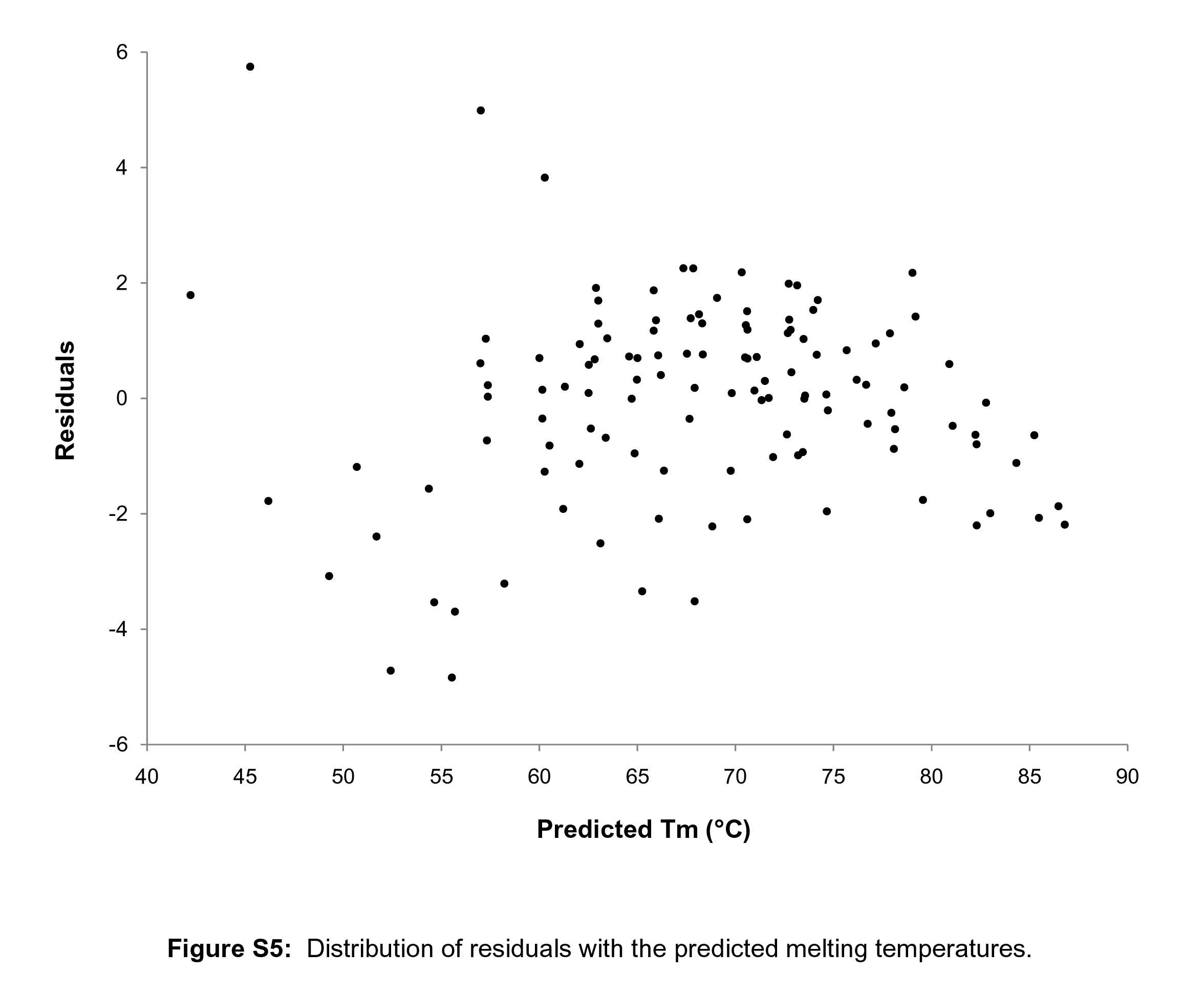

Supplement: Figure S5 — Distribution of residuals with the predicted melting temperatures. (0.37 MB TIF) [file pone.0012433.s005.tif]
