# Supplementary material for: A Phenomenological Model for Predicting Melting Temperatures of DNA Sequences
Source: PLoS One. 2010 Aug 26;5(8):e12433. doi: 10.1371/journal.pone.0012433 (PMC2928768; doi:10.1371/journal.pone.0012433)
Supplement: Text S1 — The equation to predict the melting temperature of DNA without the use of the nucleotide strand concentration. (0.02 MB DOC) [file pone.0012433.s006.doc]

The equation to predict the melting temperature of DNA without the use of the nucleotide strand concentration is:

**Tm(°C) = (7.31 x E) + [16.47 x ln (Len)] + [4.97 x ln (Conc)] – 33.81** (Equation S1)

Tm = Predicted melting temperature

E = DNA strength parameter per base

Len = Length of nucleotide sequence (number of base pairs)

Conc = [Na+] concentration of the solution (Molar)
