# Supplementary material for: A Phenomenological Model for Predicting Melting Temperatures of DNA Sequences
Source: PLoS One. 2010 Aug 26;5(8):e12433. doi: 10.1371/journal.pone.0012433 (PMC2928768; doi:10.1371/journal.pone.0012433)
Supplement: Table S1 — Experimental and predicted melting temperatures for the training dataset of 123 oligomers. (0.22 MB DOC) [file pone.0012433.s007.doc]

**Table S1:** Experimental and predicted melting temperatures for the training dataset of 123 oligomers [37]

| S. No. | Length (bp) | Salt Conc. (M) | DNA Conc. (M) | Oligonucleotide Sequence | Exp. Tm (°C) | Predicted Tm (°C) |
| --- | --- | --- | --- | --- | --- | --- |
|  | 16 | 0.12 | 0.000005 | AAAAAAAGCTTTTTTT | 44 | 42.16 |
|  | 16 | 0.12 | 0.0000074 | AACGTGAATTCTGGCA | 58.3 | 57.21 |
|  | 16 | 1 | 0.000018 | CAACTTGATATTAATA | 52 | 55.66 |
|  | 16 | 1 | 0.000100 | CATATTGGCCAATATG | 65.3 | 64.53 |
|  | 17 | 0.1 | 0.000004 | CTCGTACCTTCCGGTCC | 60.6 | 63.05 |
|  | 20 | 0.12 | 0.000005 | AAAAAAAAAGCTTTTTTTTT | 51 | 45.20 |
|  | 20 | 1 | 0.000002 | AGAAGGGAGAGAAAAAGAAA | 61.9 | 65.19 |
|  | 20 | 0.07 | 0.000002 | TATGTATATTTTGTAATCAG | 44.4 | 46.12 |
|  | 20 | 0.62 | 0.000002 | TATGTATATTTTGTAATCAG | 57.6 | 56.94 |
|  | 20 | 0.12 | 0.000002 | TTCAAGTTAAACATTCTATC | 49.5 | 50.64 |
|  | 20 | 1.02 | 0.000002 | TTCAAGTTAAACATTCTATC | 61.5 | 61.25 |
|  | 20 | 0.22 | 0.000002 | TGATTCTACCTATGTGATTT | 57.4 | 57.32 |
|  | 20 | 0.07 | 0.000002 | GAGATTGTTTCCCTTTCAAA | 49.3 | 51.64 |
|  | 20 | 0.12 | 0.000002 | GAGATTGTTTCCCTTTCAAA | 52.8 | 54.31 |
|  | 20 | 0.22 | 0.000002 | GAGATTGTTTCCCTTTCAAA | 57.6 | 57.32 |
|  | 20 | 0.62 | 0.000002 | GAGATTGTTTCCCTTTCAAA | 62.6 | 62.46 |
|  | 20 | 1.02 | 0.000002 | GAGATTGTTTCCCTTTCAAA | 65.3 | 64.93 |
|  | 20 | 0.07 | 0.000002 | CCACTATACCATCTATGTAC | 51.1 | 54.58 |
|  | 20 | 1.02 | 0.000002 | CCACTATACCATCTATGTAC | 64.4 | 67.87 |
|  | 20 | 0.22 | 0.000002 | CCATCATTGTGTCTACCTCA | 63.1 | 62.46 |
|  | 20 | 0.62 | 0.000002 | CCATCATTGTGTCTACCTCA | 67.3 | 67.60 |
|  | 20 | 1.02 | 0.000002 | CGGGACCAACTAAAGGAAAT | 68.5 | 69.70 |
|  | 20 | 0.22 | 0.000002 | TAGTGGCGATTAGATTCTGC | 64.8 | 62.83 |
|  | 20 | 1.02 | 0.000002 | TAGTGGCGATTAGATTCTGC | 71.2 | 70.44 |
|  | 20 | 0.07 | 0.000002 | AGCTGCAGTGGATGTGAGAA | 59.7 | 60.46 |
|  | 20 | 0.62 | 0.000002 | AGCTGCAGTGGATGTGAGAA | 71.3 | 71.28 |
|  | 20 | 0.07 | 0.000002 | TACTTCCAGTGCTCAGCGTA | 60.3 | 60.09 |
|  | 20 | 0.22 | 0.000002 | TACTTCCAGTGCTCAGCGTA | 67.7 | 65.77 |
|  | 20 | 0.07 | 0.000002 | CAGTGAGACAGCAATGGTCG | 59.8 | 60.09 |
|  | 20 | 0.12 | 0.000002 | CAGTGAGACAGCAATGGTCG | 63.5 | 62.76 |
|  | 20 | 0.22 | 0.000002 | CAGTGAGACAGCAATGGTCG | 67 | 65.77 |
|  | 20 | 0.62 | 0.000002 | CAGTGAGACAGCAATGGTCG | 71.1 | 70.91 |
|  | 20 | 1.02 | 0.000002 | CAGTGAGACAGCAATGGTCG | 72.5 | 73.38 |
|  | 20 | 0.62 | 0.000002 | CGAGCTTATCCCTATCCCTC | 68.5 | 70.54 |
|  | 20 | 0.22 | 0.000002 | CGTACTAGCGTTGGTCATGG | 66.6 | 66.14 |
|  | 20 | 0.07 | 0.000002 | AAGGCGAGTCAGGCTCAGTG | 64.5 | 63.40 |
|  | 20 | 1.02 | 0.000002 | AAGGCGAGTCAGGCTCAGTG | 76.3 | 76.69 |
|  | 20 | 0.12 | 0.000002 | ACCGACGACGCTGATCCGAT | 69.1 | 68.28 |
|  | 20 | 0.12 | 0.000002 | AGCAGTCCGCCACACCCTGA | 69.9 | 69.75 |
|  | 20 | 0.22 | 0.000002 | AGCAGTCCGCCACACCCTGA | 74 | 72.75 |
|  | 20 | 0.62 | 0.000002 | CAGCCTCGTTCGCACAGCCC | 77.7 | 77.89 |
|  | 20 | 1.02 | 0.000002 | GTGGTGGGCCGTGCGCTCTG | 81 | 82.93 |
|  | 20 | 0.07 | 0.000002 | GTCCACGCCCGGTGCGACGG | 70.9 | 71.85 |
|  | 22 | 0.12 | 0.0000033 | AATATATATAGAGTGCGCGCAA | 64.1 | 60.22 |
|  | 22 | 0.12 | 0.0000041 | ACTGGCGAGGATCCAGCGGTCA | 71.8 | 71.44 |
|  | 22 | 0.12 | 0.0000058 | ATAAATTAGGATCCATATAAAT | 47.7 | 52.37 |
|  | 22 | 0.12 | 0.0000053 | GCGGGCGAGGATCCAGCGCGCG | 77.2 | 78.01 |
|  | 22 | 0.12 | 0.0000059 | AACGCCGGTAGAGTGCGCGCAA | 79.1 | 72.43 |
|  | 22 | 0.15 | 0.0000011 | GATGACGCTAGCTAGCTAGGAC | 64 | 66.03 |
|  | 24 | 0.12 | 0.0000044 | AATATATATGAATTCTAATTATAA | 46.2 | 49.23 |
|  | 25 | 0.1 | 0.000001 | GTAGAATTCTTTTCCTTCTAGATCG | 62 | 56.95 |
|  | 25 | 0.07 | 0.000002 | ATAACTTTACGTGTGTGACCTATTA | 56.6 | 57.27 |
|  | 25 | 0.12 | 0.000002 | ATAACTTTACGTGTGTGACCTATTA | 60.7 | 59.94 |
|  | 25 | 0.22 | 0.000002 | ATAACTTTACGTGTGTGACCTATTA | 64.7 | 62.95 |
|  | 25 | 0.62 | 0.000002 | ATAACTTTACGTGTGTGACCTATTA | 69.6 | 68.09 |
|  | 25 | 1.02 | 0.000002 | ATAACTTTACGTGTGTGACCTATTA | 71.8 | 70.56 |
|  | 25 | 0.62 | 0.000002 | CCCTGCACTTTAACTGAATTGTTTA | 70.1 | 67.80 |
|  | 25 | 1.02 | 0.000002 | CCCTGCACTTTAACTGAATTGTTTA | 72.5 | 70.26 |
|  | 25 | 0.22 | 0.000002 | TAACCATACTGAATACCTTTTGACG | 64.3 | 62.95 |
|  | 25 | 1.02 | 0.000002 | TAACCATACTGAATACCTTTTGACG | 71.3 | 70.56 |
|  | 25 | 0.22 | 0.000002 | TCCACACGGTAGTAAAATTAGGCTT | 67.3 | 65.89 |
|  | 25 | 0.62 | 0.000002 | TCCACACGGTAGTAAAATTAGGCTT | 71.8 | 71.03 |
|  | 25 | 0.12 | 0.000002 | TTCCAAAAGGAGTTATGAGTTGCGA | 63 | 62.00 |
|  | 25 | 1.02 | 0.000002 | TTCCAAAAGGAGTTATGAGTTGCGA | 73.8 | 72.62 |
|  | 25 | 0.12 | 0.000002 | AATATCTCTCATGCGCCAAGCTACA | 65.7 | 64.94 |
|  | 25 | 0.62 | 0.000002 | AATATCTCTCATGCGCCAAGCTACA | 75.1 | 73.09 |
|  | 25 | 0.12 | 0.000002 | TAGTATATCGCAGCATCATACAGGC | 64.7 | 64.65 |
|  | 25 | 0.22 | 0.000002 | TAGTATATCGCAGCATCATACAGGC | 69.1 | 67.65 |
|  | 25 | 0.07 | 0.000002 | TGGATTCTACTCAACCTTAGTCTGG | 59 | 60.21 |
|  | 25 | 1.02 | 0.000002 | TGGATTCTACTCAACCTTAGTCTGG | 73.6 | 73.50 |
|  | 25 | 0.07 | 0.000002 | CGGAATCCATGTTACTTCGGCTATC | 60.9 | 61.97 |
|  | 25 | 0.62 | 0.000002 | CGGAATCCATGTTACTTCGGCTATC | 73.3 | 72.79 |
|  | 25 | 0.07 | 0.000002 | CTGGTCTGGATCTGAGAACTTCAGG | 62.1 | 62.56 |
|  | 25 | 0.22 | 0.000002 | CTGGTCTGGATCTGAGAACTTCAGG | 69.6 | 68.24 |
|  | 25 | 0.07 | 0.000002 | ACAGCGAATGGACCTACGTGGCCTT | 68.1 | 67.85 |
|  | 25 | 0.12 | 0.000002 | ACAGCGAATGGACCTACGTGGCCTT | 72.1 | 70.53 |
|  | 25 | 1.02 | 0.000002 | AGCAAGTCGAGCAGGGCCTACGTTT | 81.5 | 80.85 |
|  | 25 | 0.22 | 0.000002 | GCGAGCGACAGGTTACTTGGCTGAT | 74.7 | 72.65 |
|  | 25 | 0.62 | 0.000002 | AAAGGTGTCGCGGAGAGTCGTGCTG | 81.2 | 78.97 |
|  | 25 | 0.12 | 0.000002 | ATGGGTGGGAGCCTCGGTAGCAGCC | 74.5 | 74.64 |
|  | 25 | 0.07 | 0.000002 | CAGTGGGCTCCTGGGCGTGCTGGTC | 72 | 72.56 |
|  | 25 | 0.07 | 0.000002 | GCCAACTCCGTCGCCGTTCGTGCGC | 73.5 | 73.44 |
|  | 25 | 0.12 | 0.000002 | GCCAACTCCGTCGCCGTTCGTGCGC | 76.5 | 76.11 |
|  | 25 | 0.22 | 0.000002 | GCCAACTCCGTCGCCGTTCGTGCGC | 80.6 | 79.12 |
|  | 25 | 0.62 | 0.000002 | GCCAACTCCGTCGCCGTTCGTGCGC | 83.2 | 84.26 |
|  | 25 | 1.02 | 0.000002 | GCCAACTCCGTCGCCGTTCGTGCGC | 84.6 | 86.73 |
|  | 30 | 0.07 | 0.000002 | TTATGTATTAAGTTATATAGTAGTAGTAGT | 50.7 | 55.48 |
|  | 30 | 0.12 | 0.000002 | TTATGTATTAAGTTATATAGTAGTAGTAGT | 55 | 58.16 |
|  | 30 | 0.22 | 0.000002 | TTATGTATTAAGTTATATAGTAGTAGTAGT | 59.3 | 61.16 |
|  | 30 | 0.62 | 0.000002 | TTATGTATTAAGTTATATAGTAGTAGTAGT | 65.1 | 66.30 |
|  | 30 | 1.02 | 0.000002 | TTATGTATTAAGTTATATAGTAGTAGTAGT | 66.6 | 68.77 |
|  | 30 | 1.02 | 0.000002 | CTCAACTTGCGGTAAATAAATCGCTTAATC | 75.5 | 73.92 |
|  | 30 | 0.22 | 0.000002 | TATTGAGAACAAGTGTCCGATTAGCAGAAA | 69.6 | 67.29 |
|  | 30 | 0.07 | 0.000002 | GTCATACGACTGAGTGCAACATTGTTCAAA | 62.7 | 63.32 |
|  | 30 | 0.12 | 0.000002 | GTCATACGACTGAGTGCAACATTGTTCAAA | 66.8 | 66.00 |
|  | 30 | 0.22 | 0.000002 | GTCATACGACTGAGTGCAACATTGTTCAAA | 70.8 | 69.00 |
|  | 30 | 0.62 | 0.000002 | GTCATACGACTGAGTGCAACATTGTTCAAA | 75.9 | 74.14 |
|  | 30 | 1.02 | 0.000002 | GTCATACGACTGAGTGCAACATTGTTCAAA | 76.9 | 76.61 |
|  | 30 | 0.07 | 0.000002 | CCGTGCGGTGTGTACGTTTTATTCATCATA | 63.9 | 64.79 |
|  | 30 | 0.12 | 0.000002 | CCGTGCGGTGTGTACGTTTTATTCATCATA | 68.3 | 67.47 |
|  | 30 | 0.22 | 0.000002 | CCGTGCGGTGTGTACGTTTTATTCATCATA | 71.8 | 70.47 |
|  | 30 | 0.62 | 0.000002 | CCGTGCGGTGTGTACGTTTTATTCATCATA | 76.5 | 75.61 |
|  | 30 | 1.02 | 0.000002 | CCGTGCGGTGTGTACGTTTTATTCATCATA | 77.6 | 78.08 |
|  | 30 | 0.22 | 0.000002 | AGTCTGGTCTGGATCTGAGAACTTCAGGCT | 74.5 | 73.41 |
|  | 30 | 0.62 | 0.000002 | AGTCTGGTCTGGATCTGAGAACTTCAGGCT | 78.8 | 78.55 |
|  | 30 | 1.02 | 0.000002 | AGTCTGGTCTGGATCTGAGAACTTCAGGCT | 80.6 | 81.02 |
|  | 30 | 0.22 | 0.000002 | TCGGAGAAATCACTGAGCTGCCTGAGAAGA | 74.1 | 72.68 |
|  | 30 | 0.62 | 0.000002 | TCGGAGAAATCACTGAGCTGCCTGAGAAGA | 79 | 77.82 |
|  | 30 | 0.12 | 0.000002 | CTTCAACGGATCAGGTAGGACTGTGGTGGG | 71.7 | 71.63 |
|  | 30 | 1.02 | 0.000002 | CTTCAACGGATCAGGTAGGACTGTGGTGGG | 80.1 | 82.25 |
|  | 30 | 0.12 | 0.000002 | ACGCCCACAGGATTAGGCTGGCCCACATTG | 74.7 | 74.57 |
|  | 30 | 0.62 | 0.000002 | ACGCCCACAGGATTAGGCTGGCCCACATTG | 82.7 | 82.72 |
|  | 30 | 0.12 | 0.000002 | GTTATTCCGCAGTCCGATGGCAGCAGGCTC | 74.9 | 74.08 |
|  | 30 | 0.22 | 0.000002 | GTTATTCCGCAGTCCGATGGCAGCAGGCTC | 78.1 | 77.09 |
|  | 30 | 0.07 | 0.000002 | TCAGTAGGCGTGACGCAGAGCTGGCGATGG | 72.2 | 73.12 |
|  | 30 | 1.02 | 0.000002 | TCAGTAGGCGTGACGCAGAGCTGGCGATGG | 84.6 | 86.41 |
|  | 30 | 0.07 | 0.000002 | CGCGCCACGTGTGATCTACAGCCGTTCGGC | 72.7 | 74.59 |
|  | 30 | 0.62 | 0.000002 | CGCGCCACGTGTGATCTACAGCCGTTCGGC | 83.4 | 85.41 |
|  | 30 | 0.22 | 0.000002 | GACCTGACGTGGACCGCTCCTGGGCGTGGT | 81.5 | 82.23 |
|  | 30 | 0.07 | 0.000002 | CGCCGCTGCCGACTGGAGGAGCGCGGGACG | 77.8 | 79.49 |
|  | 30 | 0.12 | 0.000002 | CGCCGCTGCCGACTGGAGGAGCGCGGGACG | 81.6 | 82.17 |
|  | 30 | 0.22 | 0.000002 | CGCCGCTGCCGACTGGAGGAGCGCGGGACG | 84.6 | 85.17 |
|  | 30 | 0.62 | 0.000002 | CGCCGCTGCCGACTGGAGGAGCGCGGGACG | 87.7 | 90.31 |
