# Supplementary material for: A Phenomenological Model for Predicting Melting Temperatures of DNA Sequences
Source: PLoS One. 2010 Aug 26;5(8):e12433. doi: 10.1371/journal.pone.0012433 (PMC2928768; doi:10.1371/journal.pone.0012433)
Supplement: Table S2 — Experimental and predicted melting temperatures for the test dataset of 225 oligomers. (0.38 MB DOC) [file pone.0012433.s008.doc]

**Table S2:** Experimental and predicted melting temperatures for the test dataset of 225 oligomers [37]

| S. No. | Length (bp) | Salt Conc. (M) | DNA Conc. (M) | Oligonucleotide Sequence | Exp. Tm (°C) | Predicted Tm (°C) |
| --- | --- | --- | --- | --- | --- | --- |
|  | 16 | 0.12 | 0.000005 | AAATATAGCTATATTT | 40.4 | 44.00 |
|  | 16 | 0.12 | 0.000006 | AATATGAATTCTAATA | 41.4 | 43.24 |
|  | 16 | 0.12 | 0.000005 | ATATATAGCTATATAT | 39.1 | 44.92 |
|  | 16 | 1 | 0.000100 | GTATAACCGGTTATAC | 65.9 | 65.91 |
|  | 16 | 0.12 | 0.000044 | CGCGCGCGACTGACTG | 69.4 | 69.82 |
|  | 16 | 0.1 | 0.000004 | CGCAAAAAAAAAACGC | 54.1 | 49.79 |
|  | 17 | 0.12 | 0.000005 | AAGCTATATATATCGAT | 50.2 | 50.32 |
|  | 17 | 0.12 | 0.000004 | AATATATAGCTATCGAT | 49.7 | 50.12 |
|  | 17 | 0.12 | 0.000003 | AATATATGATCATCGAT | 50 | 49.86 |
|  | 17 | 0.12 | 0.000004 | AGATCATATATATCGAT | 49.2 | 50.12 |
|  | 18 | 0.1 | 0.000004 | CTCGTACCTTTCCGGTCC | 61.7 | 62.65 |
|  | 20 | 0.12 | 0.000007 | AAATAAATAGCTATTTATTT | 47.1 | 46.97 |
|  | 20 | 0.12 | 0.000006 | AACGCGTGAATTCTGGCCAA | 66.6 | 64.11 |
|  | 20 | 0.12 | 0.000006 | AATATATGAATTCTAATTAA | 44.7 | 46.47 |
|  | 20 | 0.12 | 0.000007 | ATAAATAAAGCTTTATTTAT | 46.2 | 46.97 |
|  | 20 | 0.12 | 0.000006 | ATATATATAGCTTTTTTTTT | 44.3 | 46.84 |
|  | 20 | 0.12 | 0.000002 | TATGTATATTTTGTAATCAG | 47.7 | 48.80 |
|  | 20 | 0.22 | 0.000002 | TATGTATATTTTGTAATCAG | 52.6 | 51.80 |
|  | 20 | 1.02 | 0.000002 | TATGTATATTTTGTAATCAG | 61.2 | 59.41 |
|  | 20 | 0.07 | 0.000002 | TTCAAGTTAAACATTCTATC | 45.7 | 47.96 |
|  | 20 | 0.22 | 0.000002 | TTCAAGTTAAACATTCTATC | 53.9 | 53.64 |
|  | 20 | 0.62 | 0.000002 | TTCAAGTTAAACATTCTATC | 59.4 | 58.78 |
|  | 20 | 0.07 | 0.000002 | TGATTCTACCTATGTGATTT | 49.1 | 51.64 |
|  | 20 | 0.12 | 0.000002 | TGATTCTACCTATGTGATTT | 53.5 | 54.31 |
|  | 20 | 0.62 | 0.000002 | TGATTCTACCTATGTGATTT | 62.3 | 62.46 |
|  | 20 | 1.02 | 0.000002 | TGATTCTACCTATGTGATTT | 64.4 | 64.93 |
|  | 20 | 0.07 | 0.000002 | ATGCAATGCTACATATTCGC | 55.2 | 56.41 |
|  | 20 | 0.12 | 0.000002 | ATGCAATGCTACATATTCGC | 59.5 | 59.09 |
|  | 20 | 0.22 | 0.000002 | ATGCAATGCTACATATTCGC | 62.9 | 62.09 |
|  | 20 | 0.62 | 0.000002 | ATGCAATGCTACATATTCGC | 67 | 67.23 |
|  | 20 | 1.02 | 0.000002 | ATGCAATGCTACATATTCGC | 68.9 | 69.70 |
|  | 20 | 0.12 | 0.000002 | CCACTATACCATCTATGTAC | 54.6 | 57.25 |
|  | 20 | 0.22 | 0.000002 | CCACTATACCATCTATGTAC | 58.4 | 60.26 |
|  | 20 | 0.62 | 0.000002 | CCACTATACCATCTATGTAC | 62.2 | 65.40 |
|  | 20 | 0.07 | 0.000002 | CCATCATTGTGTCTACCTCA | 55.6 | 56.78 |
|  | 20 | 0.12 | 0.000002 | CCATCATTGTGTCTACCTCA | 59.5 | 59.46 |
|  | 20 | 1.02 | 0.000002 | CCATCATTGTGTCTACCTCA | 68.5 | 70.07 |
|  | 20 | 0.07 | 0.000002 | CGGGACCAACTAAAGGAAAT | 53.7 | 56.41 |
|  | 20 | 0.12 | 0.000002 | CGGGACCAACTAAAGGAAAT | 57.7 | 59.09 |
|  | 20 | 0.22 | 0.000002 | CGGGACCAACTAAAGGAAAT | 61.7 | 62.09 |
|  | 20 | 0.62 | 0.000002 | CGGGACCAACTAAAGGAAAT | 66.7 | 67.23 |
|  | 20 | 0.07 | 0.000002 | TAGTGGCGATTAGATTCTGC | 57 | 57.15 |
|  | 20 | 0.12 | 0.000002 | TAGTGGCGATTAGATTCTGC | 60.6 | 59.82 |
|  | 20 | 0.62 | 0.000002 | TAGTGGCGATTAGATTCTGC | 69.1 | 67.97 |
|  | 20 | 0.12 | 0.000002 | AGCTGCAGTGGATGTGAGAA | 63.5 | 63.13 |
|  | 20 | 0.22 | 0.000002 | AGCTGCAGTGGATGTGAGAA | 67.6 | 66.14 |
|  | 20 | 1.02 | 0.000002 | AGCTGCAGTGGATGTGAGAA | 73.1 | 73.75 |
|  | 20 | 0.12 | 0.000002 | TACTTCCAGTGCTCAGCGTA | 64.4 | 62.76 |
|  | 20 | 0.62 | 0.000002 | TACTTCCAGTGCTCAGCGTA | 71.6 | 70.91 |
|  | 20 | 1.02 | 0.000002 | TACTTCCAGTGCTCAGCGTA | 73.6 | 73.38 |
|  | 20 | 0.07 | 0.000002 | CGAGCTTATCCCTATCCCTC | 56 | 59.72 |
|  | 20 | 0.12 | 0.000002 | CGAGCTTATCCCTATCCCTC | 60.2 | 62.40 |
|  | 20 | 0.22 | 0.000002 | CGAGCTTATCCCTATCCCTC | 64.1 | 65.40 |
|  | 20 | 1.02 | 0.000002 | CGAGCTTATCCCTATCCCTC | 70.3 | 73.01 |
|  | 20 | 0.07 | 0.000002 | CGTACTAGCGTTGGTCATGG | 59.6 | 60.46 |
|  | 20 | 0.12 | 0.000002 | CGTACTAGCGTTGGTCATGG | 63.1 | 63.13 |
|  | 20 | 0.62 | 0.000002 | CGTACTAGCGTTGGTCATGG | 70.5 | 71.28 |
|  | 20 | 1.02 | 0.000002 | CGTACTAGCGTTGGTCATGG | 71.1 | 73.75 |
|  | 20 | 0.12 | 0.000002 | AAGGCGAGTCAGGCTCAGTG | 67.7 | 66.07 |
|  | 20 | 0.22 | 0.000002 | AAGGCGAGTCAGGCTCAGTG | 71.4 | 69.08 |
|  | 20 | 0.62 | 0.000002 | AAGGCGAGTCAGGCTCAGTG | 75.1 | 74.22 |
|  | 20 | 0.07 | 0.000002 | ACCGACGACGCTGATCCGAT | 66 | 65.60 |
|  | 20 | 0.22 | 0.000002 | ACCGACGACGCTGATCCGAT | 72.6 | 71.28 |
|  | 20 | 0.62 | 0.000002 | ACCGACGACGCTGATCCGAT | 76.8 | 76.42 |
|  | 20 | 1.02 | 0.000002 | ACCGACGACGCTGATCCGAT | 77.3 | 78.89 |
|  | 20 | 0.07 | 0.000002 | AGCAGTCCGCCACACCCTGA | 66.5 | 67.07 |
|  | 20 | 0.62 | 0.000002 | AGCAGTCCGCCACACCCTGA | 76.9 | 77.89 |
|  | 20 | 1.02 | 0.000002 | AGCAGTCCGCCACACCCTGA | 78.5 | 80.36 |
|  | 20 | 0.07 | 0.000002 | CAGCCTCGTTCGCACAGCCC | 67.2 | 67.07 |
|  | 20 | 0.12 | 0.000002 | CAGCCTCGTTCGCACAGCCC | 70.7 | 69.75 |
|  | 20 | 0.22 | 0.000002 | CAGCCTCGTTCGCACAGCCC | 74 | 72.75 |
|  | 20 | 1.02 | 0.000002 | CAGCCTCGTTCGCACAGCCC | 78.1 | 80.36 |
|  | 20 | 0.07 | 0.000002 | GTGGTGGGCCGTGCGCTCTG | 69.2 | 69.64 |
|  | 20 | 0.12 | 0.000002 | GTGGTGGGCCGTGCGCTCTG | 72.7 | 72.32 |
|  | 20 | 0.22 | 0.000002 | GTGGTGGGCCGTGCGCTCTG | 76.2 | 75.32 |
|  | 20 | 0.62 | 0.000002 | GTGGTGGGCCGTGCGCTCTG | 79.6 | 80.46 |
|  | 20 | 0.12 | 0.000002 | GTCCACGCCCGGTGCGACGG | 73.9 | 74.52 |
|  | 20 | 0.22 | 0.000002 | GTCCACGCCCGGTGCGACGG | 77.3 | 77.53 |
|  | 20 | 0.62 | 0.000002 | GTCCACGCCCGGTGCGACGG | 79.8 | 82.67 |
|  | 20 | 1.02 | 0.000002 | GTCCACGCCCGGTGCGACGG | 81.1 | 85.14 |
|  | 20 | 0.12 | 0.000005 | ATATATATAGCTATATATAT | 44.8 | 48.14 |
|  | 21 | 0.1 | 0.000004 | GCCGAGGTCCATGTCGTACGC | 69.5 | 69.99 |
|  | 21 | 1 | 0.000003 | GCTAAAAAGAGAGAGAGATCG | 69 | 68.55 |
|  | 22 | 0.12 | 0.000006 | AAAAAAAAGAATTCTTTTTTTT | 49.9 | 46.72 |
|  | 22 | 0.12 | 0.000007 | AAATATAAGAATTCTTATATTT | 48.9 | 48.19 |
|  | 22 | 0.12 | 0.000006 | AACGCGCGTAGAGTAATTATAA | 65 | 60.42 |
|  | 22 | 0.12 | 0.000006 | AACGCGCGTAGAGTGGCCGCAA | 77.3 | 72.44 |
|  | 22 | 0.12 | 0.000006 | AATATATATAGAGTGGCCGCAA | 65.2 | 60.42 |
|  | 22 | 0.12 | 0.000005 | ACTGATTAGGATCCATATGTCA | 58.3 | 59.92 |
|  | 22 | 0.12 | 0.000007 | ATAAAAATGAATTCATTTTTAT | 52.8 | 48.19 |
|  | 22 | 0.12 | 0.000004 | ATACGCGAGGATCCAGCGCAAT | 71.1 | 68.41 |
|  | 22 | 0.12 | 0.000006 | ATATATATGAATTCATATATAT | 48.9 | 49.39 |
|  | 22 | 0.12 | 0.000004 | GCGGATTAGGATCCATATCGCG | 65.4 | 65.73 |
|  | 22 | 0.12 | 0.000005 | AATATATATAGAGTAATTATAA | 45.1 | 48.23 |
|  | 24 | 0.12 | 0.000006 | AACGCGCGTGAATTCTGGCCGCAA | 75.8 | 71.56 |
|  | 25 | 0.07 | 0.000002 | GATATAGCAAAATTCTAAGTTAATA | 49.1 | 50.51 |
|  | 25 | 0.12 | 0.000002 | GATATAGCAAAATTCTAAGTTAATA | 53.5 | 53.18 |
|  | 25 | 0.22 | 0.000002 | GATATAGCAAAATTCTAAGTTAATA | 57.7 | 56.19 |
|  | 25 | 0.62 | 0.000002 | GATATAGCAAAATTCTAAGTTAATA | 63.3 | 61.33 |
|  | 25 | 1.02 | 0.000002 | GATATAGCAAAATTCTAAGTTAATA | 66.1 | 63.80 |
|  | 25 | 0.07 | 0.000002 | GTTCTATACTCTTGAAGTTGATTAC | 52.7 | 55.21 |
|  | 25 | 0.12 | 0.000002 | GTTCTATACTCTTGAAGTTGATTAC | 56.1 | 57.89 |
|  | 25 | 0.22 | 0.000002 | GTTCTATACTCTTGAAGTTGATTAC | 60.6 | 60.89 |
|  | 25 | 0.62 | 0.000002 | GTTCTATACTCTTGAAGTTGATTAC | 66.1 | 66.03 |
|  | 25 | 1.02 | 0.000002 | GTTCTATACTCTTGAAGTTGATTAC | 67.7 | 68.50 |
|  | 25 | 0.07 | 0.000002 | CCCTGCACTTTAACTGAATTGTTTA | 57.4 | 56.98 |
|  | 25 | 0.12 | 0.000002 | CCCTGCACTTTAACTGAATTGTTTA | 61.4 | 59.65 |
|  | 25 | 0.22 | 0.000002 | CCCTGCACTTTAACTGAATTGTTTA | 65.6 | 62.66 |
|  | 25 | 0.07 | 0.000002 | TAACCATACTGAATACCTTTTGACG | 56.5 | 57.27 |
|  | 25 | 0.12 | 0.000002 | TAACCATACTGAATACCTTTTGACG | 60.2 | 59.94 |
|  | 25 | 0.62 | 0.000002 | TAACCATACTGAATACCTTTTGACG | 68.9 | 68.09 |
|  | 25 | 0.07 | 0.000002 | TCCACACGGTAGTAAAATTAGGCTT | 59.3 | 60.21 |
|  | 25 | 0.12 | 0.000002 | TCCACACGGTAGTAAAATTAGGCTT | 63.1 | 62.88 |
|  | 25 | 1.02 | 0.000002 | TCCACACGGTAGTAAAATTAGGCTT | 73.8 | 73.50 |
|  | 25 | 0.07 | 0.000002 | TTCCAAAAGGAGTTATGAGTTGCGA | 59.1 | 59.33 |
|  | 25 | 0.22 | 0.000002 | TTCCAAAAGGAGTTATGAGTTGCGA | 67.2 | 65.01 |
|  | 25 | 0.62 | 0.000002 | TTCCAAAAGGAGTTATGAGTTGCGA | 71.6 | 70.15 |
|  | 25 | 0.07 | 0.000002 | AATATCTCTCATGCGCCAAGCTACA | 62.1 | 62.27 |
|  | 25 | 0.22 | 0.000002 | AATATCTCTCATGCGCCAAGCTACA | 70.3 | 67.95 |
|  | 25 | 1.02 | 0.000002 | AATATCTCTCATGCGCCAAGCTACA | 76.5 | 75.56 |
|  | 25 | 0.07 | 0.000002 | TAGTATATCGCAGCATCATACAGGC | 61.2 | 61.97 |
|  | 25 | 0.62 | 0.000002 | TAGTATATCGCAGCATCATACAGGC | 72.8 | 72.79 |
|  | 25 | 1.02 | 0.000002 | TAGTATATCGCAGCATCATACAGGC | 75 | 75.26 |
|  | 25 | 0.12 | 0.000002 | TGGATTCTACTCAACCTTAGTCTGG | 63.1 | 62.88 |
|  | 25 | 0.22 | 0.000002 | TGGATTCTACTCAACCTTAGTCTGG | 67.1 | 65.89 |
|  | 25 | 0.62 | 0.000002 | TGGATTCTACTCAACCTTAGTCTGG | 71.3 | 71.03 |
|  | 25 | 0.12 | 0.000002 | CGGAATCCATGTTACTTCGGCTATC | 64.7 | 64.65 |
|  | 25 | 0.22 | 0.000002 | CGGAATCCATGTTACTTCGGCTATC | 68.7 | 67.65 |
|  | 25 | 1.02 | 0.000002 | CGGAATCCATGTTACTTCGGCTATC | 74.8 | 75.26 |
|  | 25 | 0.12 | 0.000002 | CTGGTCTGGATCTGAGAACTTCAGG | 65.8 | 65.24 |
|  | 25 | 0.62 | 0.000002 | CTGGTCTGGATCTGAGAACTTCAGG | 74.2 | 73.38 |
|  | 25 | 1.02 | 0.000002 | CTGGTCTGGATCTGAGAACTTCAGG | 75.6 | 75.85 |
|  | 25 | 0.22 | 0.000002 | ACAGCGAATGGACCTACGTGGCCTT | 76 | 73.53 |
|  | 25 | 0.62 | 0.000002 | ACAGCGAATGGACCTACGTGGCCTT | 79.4 | 78.67 |
|  | 25 | 1.02 | 0.000002 | ACAGCGAATGGACCTACGTGGCCTT | 81 | 81.14 |
|  | 25 | 0.07 | 0.000002 | AGCAAGTCGAGCAGGGCCTACGTTT | 68.3 | 67.56 |
|  | 25 | 0.12 | 0.000002 | AGCAAGTCGAGCAGGGCCTACGTTT | 72.6 | 70.23 |
|  | 25 | 0.22 | 0.000002 | AGCAAGTCGAGCAGGGCCTACGTTT | 76.3 | 73.24 |
|  | 25 | 0.62 | 0.000002 | AGCAAGTCGAGCAGGGCCTACGTTT | 80 | 78.38 |
|  | 25 | 0.07 | 0.000002 | GCGAGCGACAGGTTACTTGGCTGAT | 67 | 66.97 |
|  | 25 | 0.12 | 0.000002 | GCGAGCGACAGGTTACTTGGCTGAT | 70.8 | 69.65 |
|  | 25 | 0.62 | 0.000002 | GCGAGCGACAGGTTACTTGGCTGAT | 78.6 | 77.79 |
|  | 25 | 1.02 | 0.000002 | GCGAGCGACAGGTTACTTGGCTGAT | 80.1 | 80.26 |
|  | 25 | 0.07 | 0.000002 | AAAGGTGTCGCGGAGAGTCGTGCTG | 69.6 | 68.15 |
|  | 25 | 0.12 | 0.000002 | AAAGGTGTCGCGGAGAGTCGTGCTG | 73.6 | 70.82 |
|  | 25 | 0.22 | 0.000002 | AAAGGTGTCGCGGAGAGTCGTGCTG | 77.4 | 73.83 |
|  | 25 | 1.02 | 0.000002 | AAAGGTGTCGCGGAGAGTCGTGCTG | 82.4 | 81.44 |
|  | 25 | 0.07 | 0.000002 | ATGGGTGGGAGCCTCGGTAGCAGCC | 70.7 | 71.97 |
|  | 25 | 0.22 | 0.000002 | ATGGGTGGGAGCCTCGGTAGCAGCC | 78.2 | 77.65 |
|  | 25 | 0.62 | 0.000002 | ATGGGTGGGAGCCTCGGTAGCAGCC | 81.6 | 82.79 |
|  | 25 | 1.02 | 0.000002 | ATGGGTGGGAGCCTCGGTAGCAGCC | 83.4 | 85.26 |
|  | 25 | 0.12 | 0.000002 | CAGTGGGCTCCTGGGCGTGCTGGTC | 75.6 | 75.23 |
|  | 25 | 0.22 | 0.000002 | CAGTGGGCTCCTGGGCGTGCTGGTC | 79.2 | 78.24 |
|  | 25 | 0.62 | 0.000002 | CAGTGGGCTCCTGGGCGTGCTGGTC | 82.6 | 83.38 |
|  | 25 | 1.02 | 0.000002 | CAGTGGGCTCCTGGGCGTGCTGGTC | 83.4 | 85.85 |
|  | 25 | 0.07 | 0.000002 | ACGGGTCCCCGCACCGCACCGCCAG | 76.8 | 77.26 |
|  | 25 | 0.12 | 0.000002 | ACGGGTCCCCGCACCGCACCGCCAG | 79.9 | 79.94 |
|  | 25 | 0.22 | 0.000002 | ACGGGTCCCCGCACCGCACCGCCAG | 84 | 82.94 |
|  | 25 | 0.62 | 0.000002 | ACGGGTCCCCGCACCGCACCGCCAG | 87.1 | 88.08 |
|  | 25 | 1.02 | 0.000002 | ACGGGTCCCCGCACCGCACCGCCAG | 88.3 | 90.55 |
|  | 30 | 0.15 | 0.000100 | CAGCAGCAGCAGCAGCAGCAGCAGCAGCAG | 83 | 81.36 |
|  | 30 | 0.15 | 0.000100 | GACGACGACGACGACGACGACGACGACGAC | 83 | 82.10 |
|  | 30 | 0.07 | 0.000002 | ATTGATATCCTTTTCTATTCATCTTTCATT | 53.5 | 56.22 |
|  | 30 | 0.12 | 0.000002 | ATTGATATCCTTTTCTATTCATCTTTCATT | 58.3 | 58.89 |
|  | 30 | 0.22 | 0.000002 | ATTGATATCCTTTTCTATTCATCTTTCATT | 62.3 | 61.90 |
|  | 30 | 0.62 | 0.000002 | ATTGATATCCTTTTCTATTCATCTTTCATT | 68.6 | 67.04 |
|  | 30 | 1.02 | 0.000002 | ATTGATATCCTTTTCTATTCATCTTTCATT | 70.4 | 69.51 |
|  | 30 | 0.07 | 0.000002 | AAAGTACATCAACATAGAGAATTGCATTTC | 58.3 | 58.91 |
|  | 30 | 0.12 | 0.000002 | AAAGTACATCAACATAGAGAATTGCATTTC | 61.9 | 61.59 |
|  | 30 | 0.22 | 0.000002 | AAAGTACATCAACATAGAGAATTGCATTTC | 66.1 | 64.59 |
|  | 30 | 0.62 | 0.000002 | AAAGTACATCAACATAGAGAATTGCATTTC | 71.3 | 69.73 |
|  | 30 | 1.02 | 0.000002 | AAAGTACATCAACATAGAGAATTGCATTTC | 73.2 | 72.20 |
|  | 30 | 0.07 | 0.000002 | CTTAAGATATGAGAACTTCAACTAATGTGT | 56.8 | 58.42 |
|  | 30 | 0.12 | 0.000002 | CTTAAGATATGAGAACTTCAACTAATGTGT | 61.3 | 61.10 |
|  | 30 | 0.22 | 0.000002 | CTTAAGATATGAGAACTTCAACTAATGTGT | 64.9 | 64.10 |
|  | 30 | 0.62 | 0.000002 | CTTAAGATATGAGAACTTCAACTAATGTGT | 70.5 | 69.24 |
|  | 30 | 1.02 | 0.000002 | CTTAAGATATGAGAACTTCAACTAATGTGT | 71.8 | 71.71 |
|  | 30 | 0.07 | 0.000002 | CTCAACTTGCGGTAAATAAATCGCTTAATC | 60.9 | 60.63 |
|  | 30 | 0.12 | 0.000002 | CTCAACTTGCGGTAAATAAATCGCTTAATC | 64.8 | 63.30 |
|  | 30 | 0.22 | 0.000002 | CTCAACTTGCGGTAAATAAATCGCTTAATC | 68.7 | 66.31 |
|  | 30 | 0.62 | 0.000002 | CTCAACTTGCGGTAAATAAATCGCTTAATC | 74.4 | 71.45 |
|  | 30 | 0.07 | 0.000002 | TATTGAGAACAAGTGTCCGATTAGCAGAAA | 61.3 | 61.61 |
|  | 30 | 0.12 | 0.000002 | TATTGAGAACAAGTGTCCGATTAGCAGAAA | 65.4 | 64.28 |
|  | 30 | 0.62 | 0.000002 | TATTGAGAACAAGTGTCCGATTAGCAGAAA | 74.8 | 72.43 |
|  | 30 | 1.02 | 0.000002 | TATTGAGAACAAGTGTCCGATTAGCAGAAA | 76.4 | 74.90 |
|  | 30 | 0.07 | 0.000002 | AACCTGCAACATGGAGTTTTTGTCTCATGC | 64.5 | 64.79 |
|  | 30 | 0.12 | 0.000002 | AACCTGCAACATGGAGTTTTTGTCTCATGC | 68.3 | 67.47 |
|  | 30 | 0.22 | 0.000002 | AACCTGCAACATGGAGTTTTTGTCTCATGC | 72.5 | 70.47 |
|  | 30 | 0.62 | 0.000002 | AACCTGCAACATGGAGTTTTTGTCTCATGC | 77.7 | 75.61 |
|  | 30 | 1.02 | 0.000002 | AACCTGCAACATGGAGTTTTTGTCTCATGC | 78.7 | 78.08 |
|  | 30 | 0.07 | 0.000002 | GTTCACGTCCGAAAGCTCGAAAAAGGATAC | 64.3 | 65.04 |
|  | 30 | 0.12 | 0.000002 | GTTCACGTCCGAAAGCTCGAAAAAGGATAC | 68.2 | 67.71 |
|  | 30 | 0.22 | 0.000002 | GTTCACGTCCGAAAGCTCGAAAAAGGATAC | 72.1 | 70.72 |
|  | 30 | 0.62 | 0.000002 | GTTCACGTCCGAAAGCTCGAAAAAGGATAC | 77.1 | 75.86 |
|  | 30 | 1.02 | 0.000002 | GTTCACGTCCGAAAGCTCGAAAAAGGATAC | 78.7 | 78.33 |
|  | 30 | 0.07 | 0.000002 | AGTCTGGTCTGGATCTGAGAACTTCAGGCT | 66.3 | 67.73 |
|  | 30 | 0.12 | 0.000002 | AGTCTGGTCTGGATCTGAGAACTTCAGGCT | 70.4 | 70.41 |
|  | 30 | 0.07 | 0.000002 | TCGGAGAAATCACTGAGCTGCCTGAGAAGA | 66.3 | 67.00 |
|  | 30 | 0.12 | 0.000002 | TCGGAGAAATCACTGAGCTGCCTGAGAAGA | 70.4 | 69.67 |
|  | 30 | 1.02 | 0.000002 | TCGGAGAAATCACTGAGCTGCCTGAGAAGA | 80.9 | 80.29 |
|  | 30 | 0.07 | 0.000002 | CTTCAACGGATCAGGTAGGACTGTGGTGGG | 67.6 | 68.96 |
|  | 30 | 0.22 | 0.000002 | CTTCAACGGATCAGGTAGGACTGTGGTGGG | 74.7 | 74.64 |
|  | 30 | 0.62 | 0.000002 | CTTCAACGGATCAGGTAGGACTGTGGTGGG | 78.8 | 79.78 |
|  | 30 | 0.07 | 0.000002 | ACGCCCACAGGATTAGGCTGGCCCACATTG | 71.3 | 71.90 |
|  | 30 | 0.22 | 0.000002 | ACGCCCACAGGATTAGGCTGGCCCACATTG | 78.5 | 77.58 |
|  | 30 | 1.02 | 0.000002 | ACGCCCACAGGATTAGGCTGGCCCACATTG | 84 | 85.19 |
|  | 30 | 0.07 | 0.000002 | GTTATTCCGCAGTCCGATGGCAGCAGGCTC | 70.6 | 71.41 |
|  | 30 | 0.62 | 0.000002 | GTTATTCCGCAGTCCGATGGCAGCAGGCTC | 82.4 | 82.23 |
|  | 30 | 1.02 | 0.000002 | GTTATTCCGCAGTCCGATGGCAGCAGGCTC | 84.1 | 84.70 |
|  | 30 | 0.12 | 0.000002 | TCAGTAGGCGTGACGCAGAGCTGGCGATGG | 75.7 | 75.80 |
|  | 30 | 0.22 | 0.000002 | TCAGTAGGCGTGACGCAGAGCTGGCGATGG | 79.3 | 78.80 |
|  | 30 | 0.62 | 0.000002 | TCAGTAGGCGTGACGCAGAGCTGGCGATGG | 83.3 | 83.94 |
|  | 30 | 0.12 | 0.000002 | CGCGCCACGTGTGATCTACAGCCGTTCGGC | 76.3 | 77.27 |
|  | 30 | 0.22 | 0.000002 | CGCGCCACGTGTGATCTACAGCCGTTCGGC | 79.5 | 80.27 |
|  | 30 | 1.02 | 0.000002 | CGCGCCACGTGTGATCTACAGCCGTTCGGC | 84.5 | 87.88 |
|  | 30 | 0.07 | 0.000002 | GACCTGACGTGGACCGCTCCTGGGCGTGGT | 74.4 | 76.55 |
|  | 30 | 0.12 | 0.000002 | GACCTGACGTGGACCGCTCCTGGGCGTGGT | 78.4 | 79.23 |
|  | 30 | 0.62 | 0.000002 | GACCTGACGTGGACCGCTCCTGGGCGTGGT | 85.2 | 87.37 |
|  | 30 | 1.02 | 0.000002 | GACCTGACGTGGACCGCTCCTGGGCGTGGT | 86.4 | 89.84 |
|  | 30 | 0.07 | 0.000002 | GCCCCTCCACTGGCCGACGGCAGCAGGCTC | 76.3 | 78.51 |
|  | 30 | 0.12 | 0.000002 | GCCCCTCCACTGGCCGACGGCAGCAGGCTC | 79.8 | 81.19 |
|  | 30 | 0.22 | 0.000002 | GCCCCTCCACTGGCCGACGGCAGCAGGCTC | 83.6 | 84.19 |
|  | 30 | 0.62 | 0.000002 | GCCCCTCCACTGGCCGACGGCAGCAGGCTC | 87.1 | 89.33 |
|  | 30 | 1.02 | 0.000002 | GCCCCTCCACTGGCCGACGGCAGCAGGCTC | 87.7 | 91.80 |
|  | 30 | 1.02 | 0.000002 | CGCCGCTGCCGACTGGAGGAGCGCGGGACG | 88.6 | 92.78 |
