# Supplementary material for: A Phenomenological Model for Predicting Melting Temperatures of DNA Sequences
Source: PLoS One. 2010 Aug 26;5(8):e12433. doi: 10.1371/journal.pone.0012433 (PMC2928768; doi:10.1371/journal.pone.0012433)
Supplement: Table S3 — Experimental and predicted melting temperatures for a dataset of 15-mers. (0.16 MB DOC) [file pone.0012433.s009.doc]

**Table S3:** Experimental and predicted melting temperatures for a dataset of 15-mers [38]

| S. No. | Length (bp) | Salt Conc. (M) | DNA Conc. (M) | Oligonucleotide Sequence | Exp. Tm (°C) | Predicted Tm (°C) |
| --- | --- | --- | --- | --- | --- | --- |
|  | 15 | 0.07 | 0.000002 | TACTAACATTAACTA | 35.3 | 41.26 |
|  | 15 | 0.12 | 0.000002 | TACTAACATTAACTA | 40.4 | 43.93 |
|  | 15 | 0.22 | 0.000002 | TACTAACATTAACTA | 44.1 | 46.94 |
|  | 15 | 0.62 | 0.000002 | TACTAACATTAACTA | 49.3 | 52.08 |
|  | 15 | 1.02 | 0.000002 | TACTAACATTAACTA | 51.1 | 54.55 |
|  | 15 | 0.07 | 0.000002 | ATACTTACTGATTAG | 38.1 | 43.22 |
|  | 15 | 0.12 | 0.000002 | ATACTTACTGATTAG | 41.4 | 45.89 |
|  | 15 | 0.22 | 0.000002 | ATACTTACTGATTAG | 45 | 48.90 |
|  | 15 | 0.62 | 0.000002 | ATACTTACTGATTAG | 49.9 | 54.04 |
|  | 15 | 1.02 | 0.000002 | ATACTTACTGATTAG | 51.5 | 56.51 |
|  | 15 | 0.07 | 0.000002 | GTACACTGTCTTATA | 41 | 46.65 |
|  | 15 | 0.12 | 0.000002 | GTACACTGTCTTATA | 44.8 | 49.32 |
|  | 15 | 0.22 | 0.000002 | GTACACTGTCTTATA | 48.3 | 52.33 |
|  | 15 | 0.62 | 0.000002 | GTACACTGTCTTATA | 52.9 | 57.47 |
|  | 15 | 1.02 | 0.000002 | GTACACTGTCTTATA | 54.8 | 59.94 |
|  | 15 | 0.07 | 0.000002 | GTATGAGAGACTTTA | 39.9 | 45.67 |
|  | 15 | 0.12 | 0.000002 | GTATGAGAGACTTTA | 44.2 | 48.34 |
|  | 15 | 0.22 | 0.000002 | GTATGAGAGACTTTA | 47.9 | 51.35 |
|  | 15 | 0.62 | 0.000002 | GTATGAGAGACTTTA | 53.3 | 56.49 |
|  | 15 | 1.02 | 0.000002 | GTATGAGAGACTTTA | 55.4 | 58.96 |
|  | 15 | 0.07 | 0.000002 | TTCTACCTATGTGAT | 40.6 | 47.63 |
|  | 15 | 0.12 | 0.000002 | TTCTACCTATGTGAT | 44.6 | 50.30 |
|  | 15 | 0.22 | 0.000002 | TTCTACCTATGTGAT | 48.1 | 53.31 |
|  | 15 | 0.62 | 0.000002 | TTCTACCTATGTGAT | 52.3 | 58.45 |
|  | 15 | 1.02 | 0.000002 | TTCTACCTATGTGAT | 53.7 | 60.92 |
|  | 15 | 0.07 | 0.000002 | AGTAGTAATCACACC | 44.3 | 50.08 |
|  | 15 | 0.12 | 0.000002 | AGTAGTAATCACACC | 47.8 | 52.75 |
|  | 15 | 0.22 | 0.000002 | AGTAGTAATCACACC | 51.6 | 55.76 |
|  | 15 | 0.62 | 0.000002 | AGTAGTAATCACACC | 56.2 | 60.90 |
|  | 15 | 1.02 | 0.000002 | AGTAGTAATCACACC | 57.1 | 63.37 |
|  | 15 | 0.07 | 0.000002 | ATCGTCTCGGGTATAA | 45.5 | 53.51 |
|  | 15 | 0.12 | 0.000002 | ATCGTCTCGGGTATAA | 49.4 | 56.18 |
|  | 15 | 0.22 | 0.000002 | ATCGTCTCGGGTATAA | 52.9 | 59.19 |
|  | 15 | 0.62 | 0.000002 | ATCGTCTCGGGTATAA | 57.4 | 64.33 |
|  | 15 | 1.02 | 0.000002 | ATCGTCTCGGGTATAA | 58.6 | 66.80 |
|  | 15 | 0.07 | 0.000002 | ACGACAGGTTTACCA | 47.8 | 53.51 |
|  | 15 | 0.12 | 0.000002 | ACGACAGGTTTACCA | 51.2 | 56.18 |
|  | 15 | 0.22 | 0.000002 | ACGACAGGTTTACCA | 55.5 | 59.19 |
|  | 15 | 0.62 | 0.000002 | ACGACAGGTTTACCA | 59.8 | 64.33 |
|  | 15 | 1.02 | 0.000002 | ACGACAGGTTTACCA | 61.3 | 66.80 |
|  | 15 | 0.07 | 0.000002 | CTTTCATGTCCGCAT | 49.9 | 52.04 |
|  | 15 | 0.12 | 0.000002 | CTTTCATGTCCGCAT | 53.9 | 54.71 |
|  | 15 | 0.22 | 0.000002 | CTTTCATGTCCGCAT | 57.1 | 57.72 |
|  | 15 | 0.62 | 0.000002 | CTTTCATGTCCGCAT | 61.4 | 62.86 |
|  | 15 | 1.02 | 0.000002 | CTTTCATGTCCGCAT | 62.8 | 65.33 |
|  | 15 | 0.07 | 0.000002 | TGGATGTGTGAACAC | 46.5 | 52.53 |
|  | 15 | 0.12 | 0.000002 | TGGATGTGTGAACAC | 51.6 | 55.20 |
|  | 15 | 0.22 | 0.000002 | TGGATGTGTGAACAC | 54.6 | 58.21 |
|  | 15 | 0.62 | 0.000002 | TGGATGTGTGAACAC | 59.1 | 63.35 |
|  | 15 | 1.02 | 0.000002 | TGGATGTGTGAACAC | 60.4 | 65.82 |
|  | 15 | 0.07 | 0.000002 | ACCCCGCAATACATG | 51.3 | 55.47 |
|  | 15 | 0.12 | 0.000002 | ACCCCGCAATACATG | 55.2 | 58.14 |
|  | 15 | 0.22 | 0.000002 | ACCCCGCAATACATG | 58.5 | 61.15 |
|  | 15 | 0.62 | 0.000002 | ACCCCGCAATACATG | 62.4 | 66.29 |
|  | 15 | 1.02 | 0.000002 | ACCCCGCAATACATG | 62.9 | 68.76 |
|  | 15 | 0.07 | 0.000002 | GCAGTGGATGTGAGA | 51.2 | 54.98 |
|  | 15 | 0.12 | 0.000002 | GCAGTGGATGTGAGA | 54.8 | 57.65 |
|  | 15 | 0.22 | 0.000002 | GCAGTGGATGTGAGA | 58 | 60.66 |
|  | 15 | 0.62 | 0.000002 | GCAGTGGATGTGAGA | 61.7 | 65.80 |
|  | 15 | 1.02 | 0.000002 | GCAGTGGATGTGAGA | 63.3 | 68.27 |
|  | 15 | 0.07 | 0.000002 | GGTCCTTACTTGGTG | 47.8 | 53.02 |
|  | 15 | 0.12 | 0.000002 | GGTCCTTACTTGGTG | 51.6 | 55.69 |
|  | 15 | 0.22 | 0.000002 | GGTCCTTACTTGGTG | 55.1 | 58.70 |
|  | 15 | 0.62 | 0.000002 | GGTCCTTACTTGGTG | 59.1 | 63.84 |
|  | 15 | 1.02 | 0.000002 | GGTCCTTACTTGGTG | 60.3 | 66.31 |
|  | 15 | 0.07 | 0.000002 | CGCCTCATGCTCATC | 52.8 | 56.45 |
|  | 15 | 0.12 | 0.000002 | CGCCTCATGCTCATC | 56.7 | 59.12 |
|  | 15 | 0.22 | 0.000002 | CGCCTCATGCTCATC | 60.1 | 62.13 |
|  | 15 | 0.62 | 0.000002 | CGCCTCATGCTCATC | 63.6 | 67.27 |
|  | 15 | 1.02 | 0.000002 | CGCCTCATGCTCATC | 65.8 | 69.74 |
|  | 15 | 0.07 | 0.000002 | AAATAGCCGGGCCGC | 59 | 61.35 |
|  | 15 | 0.12 | 0.000002 | AAATAGCCGGGCCGC | 62.2 | 64.02 |
|  | 15 | 0.22 | 0.000002 | AAATAGCCGGGCCGC | 65.3 | 67.03 |
|  | 15 | 0.62 | 0.000002 | AAATAGCCGGGCCGC | 69 | 72.17 |
|  | 15 | 1.02 | 0.000002 | AAATAGCCGGGCCGC | 70.4 | 74.64 |
|  | 15 | 0.07 | 0.000002 | CCAGCCAGTCTCTCC | 54.1 | 58.41 |
|  | 15 | 0.12 | 0.000002 | CCAGCCAGTCTCTCC | 58 | 61.08 |
|  | 15 | 0.22 | 0.000002 | CCAGCCAGTCTCTCC | 61.5 | 64.09 |
|  | 15 | 0.62 | 0.000002 | CCAGCCAGTCTCTCC | 65.1 | 69.23 |
|  | 15 | 1.02 | 0.000002 | CCAGCCAGTCTCTCC | 66.7 | 71.70 |
|  | 15 | 0.07 | 0.000002 | GACGACAAGACCGCG | 57.9 | 59.39 |
|  | 15 | 0.12 | 0.000002 | GACGACAAGACCGCG | 61.5 | 62.06 |
|  | 15 | 0.22 | 0.000002 | GACGACAAGACCGCG | 64.4 | 65.07 |
|  | 15 | 0.62 | 0.000002 | GACGACAAGACCGCG | 67.6 | 70.21 |
|  | 15 | 1.02 | 0.000002 | GACGACAAGACCGCG | 68.6 | 72.68 |
|  | 15 | 0.07 | 0.000002 | CAGCCTCGTCGCAGC | 60.8 | 62.33 |
|  | 15 | 0.12 | 0.000002 | CAGCCTCGTCGCAGC | 64.1 | 65.00 |
|  | 15 | 0.22 | 0.000002 | CAGCCTCGTCGCAGC | 67.4 | 68.01 |
|  | 15 | 0.62 | 0.000002 | CAGCCTCGTCGCAGC | 70.1 | 73.15 |
|  | 15 | 1.02 | 0.000002 | CAGCCTCGTCGCAGC | 72 | 75.62 |
|  | 15 | 0.07 | 0.000002 | CTCGCGGTCGAAGCG | 61.5 | 61.35 |
|  | 15 | 0.12 | 0.000002 | CTCGCGGTCGAAGCG | 64.6 | 64.02 |
|  | 15 | 0.22 | 0.000002 | CTCGCGGTCGAAGCG | 67.1 | 67.03 |
|  | 15 | 0.62 | 0.000002 | CTCGCGGTCGAAGCG | 70 | 72.17 |
|  | 15 | 1.02 | 0.000002 | CTCGCGGTCGAAGCG | 70.7 | 74.64 |
|  | 15 | 0.07 | 0.000002 | GCGTCGGTCCGGGCT | 64.9 | 67.23 |
|  | 15 | 0.12 | 0.000002 | GCGTCGGTCCGGGCT | 67.7 | 69.90 |
|  | 15 | 0.22 | 0.000002 | GCGTCGGTCCGGGCT | 70.5 | 72.91 |
|  | 15 | 0.62 | 0.000002 | GCGTCGGTCCGGGCT | 73.9 | 78.05 |
|  | 15 | 1.02 | 0.000002 | GCGTCGGTCCGGGCT | 74.1 | 80.52 |
