# Supplementary material for: A Phenomenological Model for Predicting Melting Temperatures of DNA Sequences
Source: PLoS One. 2010 Aug 26;5(8):e12433. doi: 10.1371/journal.pone.0012433 (PMC2928768; doi:10.1371/journal.pone.0012433)
Supplement: Table S4 — Experimental and predicted melting temperatures for 40 base pair long oligonucleotide sequences. (0.03 MB DOC) [file pone.0012433.s010.doc]

**Table S4:** Experimental and predicted melting temperatures for 40 base pair long oligonucleotide sequences [38]

| S. No. | Salt Conc. (M) | DNA Conc. (M) | Sequence | Exp. Tm (°C) | Predicted Tm (°C) |
| --- | --- | --- | --- | --- | --- |
| 1. | 1.021 | 0.000002 | GCAATAGAAAGAGGAAATAATAGTTTTATATTCGACCTAG | 75.4 | 76.39 |
| 2. | 0.22 | 0.000002 | GCAATAGAAAGAGGAAATAATAGTTTTATATTCGACCTAG | 68 | 68.78 |
| 3. | 1.021 | 0.000002 | AGCTGACGCCAAGTCCAAATCTAACCACATGCAAGACACA | 84.5 | 85.77 |
| 4. | 0.22 | 0.000002 | AGCTGACGCCAAGTCCAAATCTAACCACATGCAAGACACA | 74.5 | 75.11 |
| 5. | 0.69 | 0.000002 | GTCCGCATCCCGAGAGCCATGTGGTGACCCTGCGCCGCAC | 78.7 | 81.59 |
| 6. | 0.621 | 0.000002 | GTCCGCATCCCGAGAGCCATGTGGTGACCCTGCGCCGCAC | 90.1 | 92.49 |
