# Supplementary material for: A Phenomenological Model for Predicting Melting Temperatures of DNA Sequences
Source: PLoS One. 2010 Aug 26;5(8):e12433. doi: 10.1371/journal.pone.0012433 (PMC2928768; doi:10.1371/journal.pone.0012433)
Supplement: Table S5 — Analysis of variance for the regression equation (1) derived from the training dataset. (0.03 MB DOC) [file pone.0012433.s011.doc]

**Table S5:** Analysis of variance for the regression equation (1) derived from the training dataset

| ANOVA |  |  |  |  |  |
| --- | --- | --- | --- | --- | --- |
|  | D.F. | SS | MS | F | P |
| Regression | 4 | 10525.66 | 2631.415 | 764.2996 | 2.5035E-83 |
| Residual | 118 | 406.2634 | 3.44291 |  |  |
| Total | 122 | 10931.92 |  |  |  |

where D.F. = Degrees of freedom

SS = Sum of squares

MS = Mean squares

F = F-statistic

P = p-value
